# Application of Alanine Scanning to Determination of Amino Acids Essential for Peptide Adsorption at the Solid/Solution Interface and Binding to the Receptor: Surface-Enhanced Raman/Infrared Spectroscopy versus Bioactivity Assays

**DOI:** 10.1021/acs.jmedchem.1c00397

**Published:** 2021-06-10

**Authors:** Edyta Proniewicz, Grzegorz Burnat, Helena Domin, Izabela Małuch, Marta Makowska, Adam Prahl

**Affiliations:** † Faculty of Foundry Engineering, AGH University of Science and Technology, 30-059 Krakow, Poland; ‡ Maj Institute of Pharmacology, Polish Academy of Sciences, Department of Neurobiology, 31-343 Kraków, 12 Smętna Street, Poland; § Faculty of Chemistry, University of Gdansk, Wita Stwosza 63, 80-308 Gdansk, Poland

## Abstract

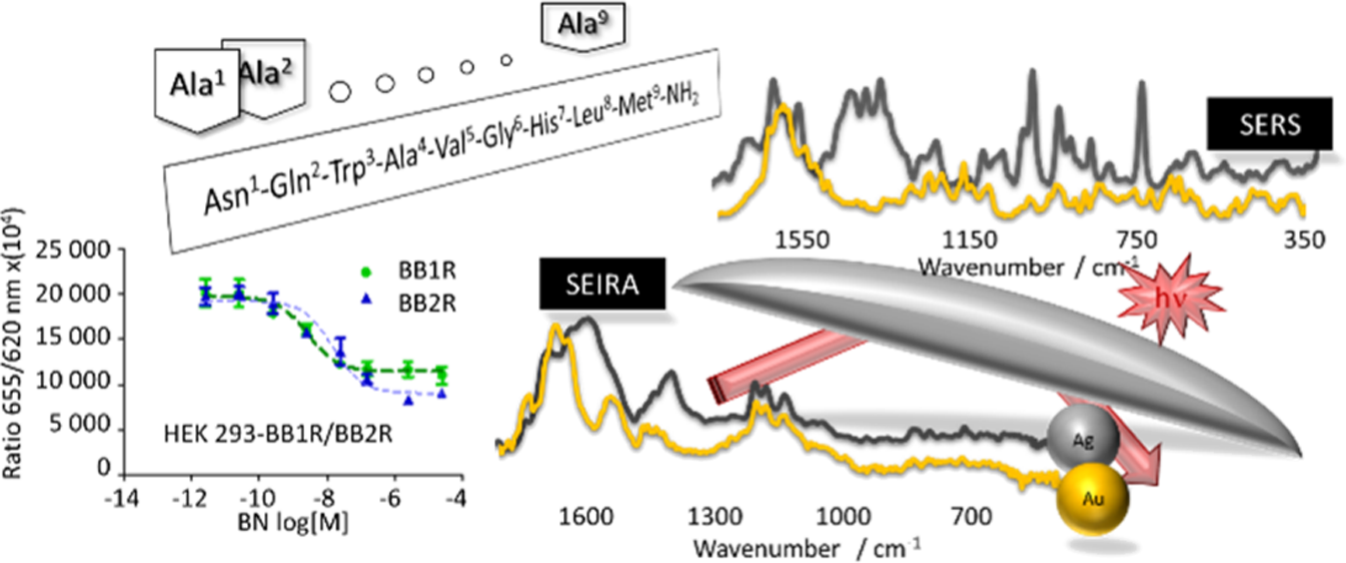

The article describes the application of the alanine-scanning technique used in combination with Raman, surface-enhanced Raman, attenuated total reflection Fourier transform infrared, and surface-enhanced infrared absorption (SEIRA) spectroscopies, which allowed defining the role of individual amino acid residues in the *C*-terminal 6–14 fragment of the bombesin chain (BN^6–14^) on the path of its adsorption on the surface of Ag (AgNPs) and Au nanoparticles (AuNPs). A reliable analysis of the SEIRA spectra of these peptides was possible, thanks to a curve fitting of these spectra. By combining alanine-scanning with biological activity studies using cell lines overexpressing bombesin receptors and the intracellular inositol monophosphate assay, it was possible to determine which peptide side chains play a significant role in binding a peptide to membrane-bound G protein-coupled receptors (GPCRs). Based on the analysis of spectral profiles and bioactivity results, conclusions for the specific peptide–metal and peptide–GPCR interactions were drawn and compared.

## Introduction

Vibrational spectroscopy [infrared absorption (IR) and Raman (RS)] is a widely used, reliable, and powerful method for studying conformational changes and molecular interactions and for unambiguously identifying and characterizing various molecules by their vibrational fingerprint. While IR is well suited for the study of polar bonds (e.g., O–H or N–H) and is mainly used to identify functional groups of molecules, the RS method is invaluable for the study of bonds formed between carbon and sulfur atoms (e.g., C–C, C=C, S–S, S–C, etc.) and enables the identification of skeletal structures. However, both methods in the conventional form do not provide sufficient sensitivity for trace concentrations and thin molecular layers (usually a few pmol/cm^2^) since most (bio)organic molecules absorb radiation in the mid-infrared range (2.5–25 μm) relatively poorly and do not scatter electromagnetic radiation effectively. This leads to a limitation of the application range of vibrational spectroscopy based on the detection of chemical traces (food safety, detection of hazardous substances, or biosensors). To overcome these limitations, surface-enhanced techniques of this method, for example, surface-enhanced Raman spectroscopy (SERS) and surface-enhanced infrared absorption spectroscopy (SEIRA), have been developed and used. These use highly concentrated fields in the vicinity of resonantly excited plasmonic structures,^1,2^ which provide signal enhancement typically of 10^1^ to 10^3^ over the signal from conventional transmission or reflection experiments (SEIRA)^3^ and of 10^3^ to 10^10^ over the signal from the classical Raman effect (SERS).^4^ The surface-enhanced techniques also overcome other limitations: since the energy of the incident radiation is very low, they minimize the risk of sample destruction or damage and can be successfully used for the study of biological materials^5^ and, in the case of infrared spectroscopy, where the extremely high IR absorption of water prevents the direct use of the water medium in IR measurements, allow measurements in aqueous solutions since the enrichment of the sample along the metal surface reduces the water content in the observed volume.

For SERS studies, noble metals (Ag,^6,7^ Au,^8,9^ and less frequently Cu,^10,11^ or other metals^12−14^) are mainly used in the form of nanostructures or thin films. The SEIRA effect is mainly studied on chemical- and vapor-deposited island layers, nanoparticle-decorated layers, and periodic array-based substrates of Ag and Au.^15,16^ Metal layers consist of growing and converging isolated particles that ultimately form a continuous layer. During this process, the signal from the adsorbate is strongly enhanced until the percolation threshold is reached (or close to it), and then, the signal strength decreases until it disappears completely after the formation of a continuous layer.^17^ The solution in this situation is the use of metal sols, which are also important for other reasons, for example, they are relatively fast, easy, and inexpensive to obtain; they allow for reproducibility of signal enhancement, thanks to synthesis procedures that ensure low dispersion of the nanoparticle diameter in the sol and do not require strict topological control; they can be used in the transmission mode without complicated optical systems, and the sample is attached to the surface of the colloidal nanoparticles before measurement. The latter advantage is particularly important in the context of the development of hybrid bio-devices (biomolecules associated with the substrate that actuates them).

Despite numerous studies on biosensors using SERS or SEIRA, there are still too many unknowns that prevent the routine use of these techniques in biology and medicine. In recent years, our research has aimed to demonstrate that SERS and now SEIRA can be used as potential tools to predict the biochemical activity of some neuropeptides.^6,18,19^ In other words, we seek to show that SERS/SEIRA techniques can be useful to identify the most important amino acid residues involved in substrate–receptor interactions in systems where biological studies are difficult or do not lead to the unequivocal identification of the molecular fragments responsible for the biological activity of the peptide.

This manuscript provides innovative insights into the biological applications of SERS/SEIRA in combination with the alanine-scanning approach, which is commonly used to determine the impact of a particular amino acid residue on protein stability or bioactivity. In addition, we performed biological activity studies using cell lines overexpressing bombesin receptors and the intracellular inositol monophosphate assay to determine which peptide side-chains play a significant role in the (bio)activity of this peptide.

To determine the biological activity of a compound, methods are commonly used that employ cell lines that overexpress the desired receptor protein, while the ideal host cells lack expression of the protein of interest. One of the most common methods is cells derived from the human embryonic kidney (HEK-293), which are widely used as host cells in bioengineering. This cell line owes its popularity to its simple maintenance methods and good transfection efficiency.^20−22^

The second piece of the puzzle is the assay to determine receptor activity. For membrane-bound G protein-coupled receptors (GPCRs), activity is usually determined by measuring the production of a second messenger such as cyclic adenosine monophosphate (cAMP) or calcium ions (Ca^2+^), depending on which type of G protein the receptor is coupled to.^23,24^ The GPCRs coupled to the Gs and Gi subtypes affect the production of cAMP, whereas the Gq subtype triggers the release of Ca^2+^ from internal storage by *d*-myo-inositol 1,4,5-trisphosphate (IP3), a product of phospholipase C β (PLC-β) activity.^25^ The release of calcium from internal storage is a relatively rapid event that is inhibited by the degradation of IP3. One of the degradation products of IP3 is the *d*-myo-inositol monophosphates (IP1), which are used to determine the activity of receptors coupled to Gq proteins.^22^

The choice of a biological system such as a fragment containing amino acids at positions 6–14 from the bombesin sequence (BN^6–14^; Asn-Gln-Trp-Ala-Val-Gly-His-Leu-Met-NH_2_) was dictated by the facts that it is a fully active fragment of this neurotransmitter known as tumor growth factor^26^ and is a ligand of GPCRs overexpressed on the surface of many malignancies.^27,28^ These facts make these receptors (when interacting with their ligands conjugated to metal nanoparticles) potentially available as receptor-positive tumor markers in the early diagnosis of tissue damage and anti-cancer therapy.^29,30^

## Results and Discussion

### SERS Spectra Analysis

**Figure 1:**
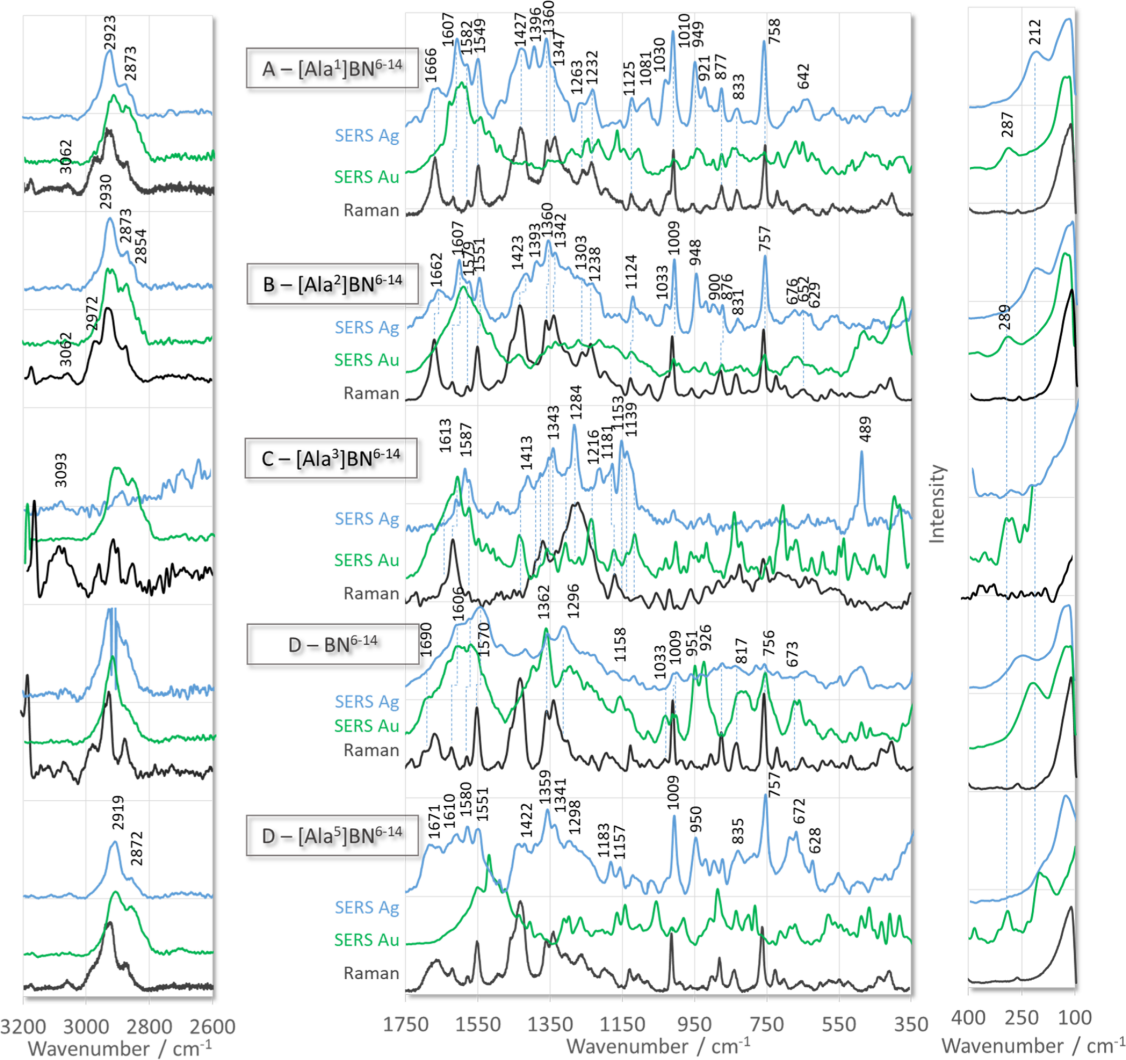
Raman (black line traces) and SERS spectra of [Ala^X^]BN^6–14^ (where X denotes amino acids at positions 1–5 of BN^6–14^ sequence) adsorbed on the surface of AgNPs (blue line traces) and AuNPs (green line traces).

**Figure 2:**
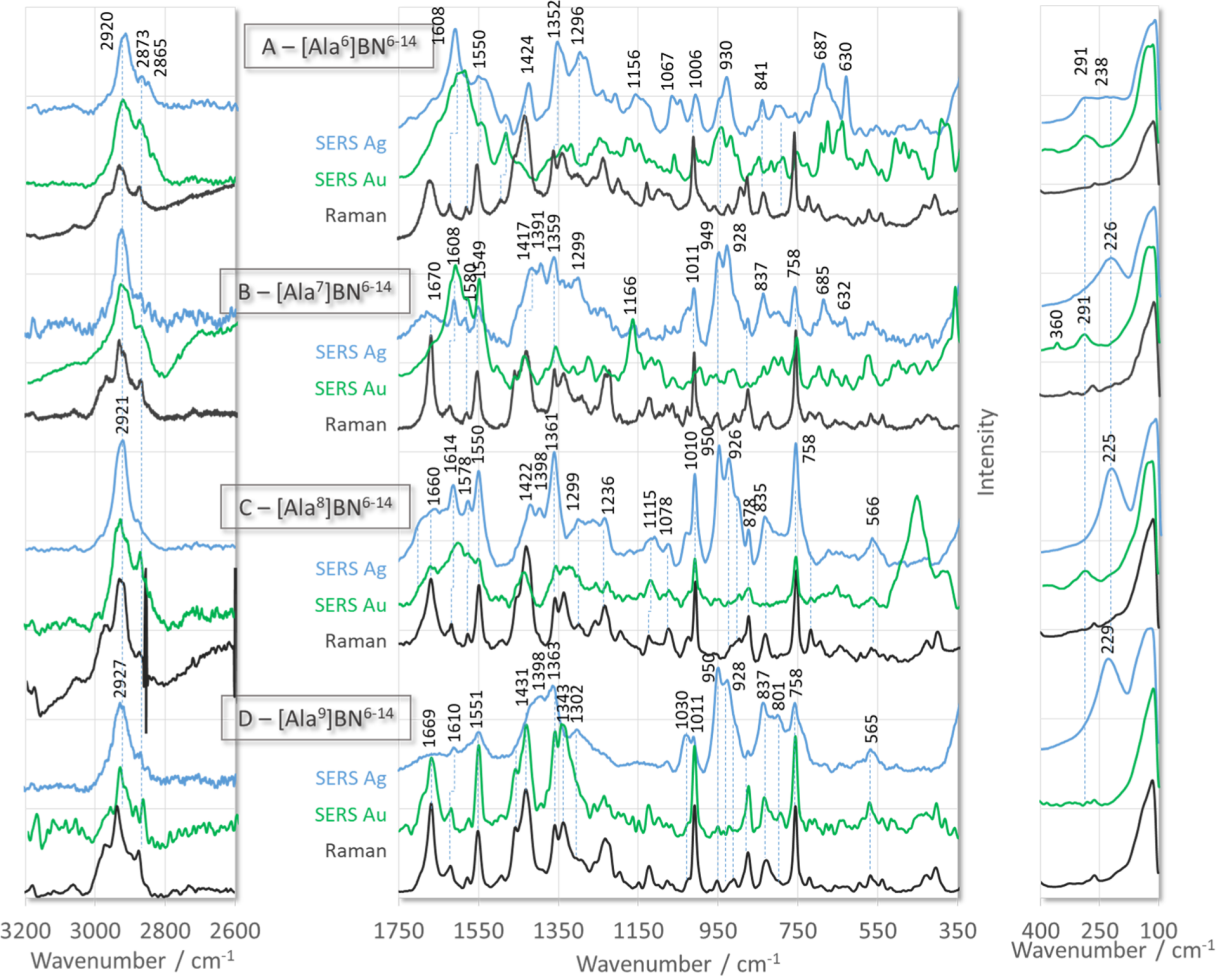
Raman (black line traces) and SERS spectra of [Ala^X^]BN^6–14^ (where X denotes amino acids at positions 6–9 of BN^6–14^ sequence) adsorbed on the surface of AgNPs (blue line traces) and AuNPs (green line traces).

Figures 1 and 2 show the SERS spectra of nine [Ala^X^]BN^6–14^ analogues immobilized on the surface of AgNPs and AuNPs (Table 1, see Supporting Information for SMILES). Raman spectra of these peptides are also included in these figures to highlight the changes between the SERS and Raman spectra. As can be seen, the spectra shown contain several often overlapping bands due to the vibrations of those molecular fragments that are on or near the surface of the metal substrate. The side-chains of the aromatic amino acids have the greatest affinity for the metal surface; therefore, many of the observed bands can be assigned to normal vibrations based on the characteristic vibrations of the aromatic rings, based on the assignment given for BN fragments of varying lengths.^31^

**Table 1:** Sequences of Studied Bombesin Analogues

symbol of peptide	sequence
[Ala^1^]BN^6–14^	Ala-Gln-Trp-Ala-Val-Gly-His-Leu-Met-NH_2_
[Ala^2^]BN^6–14^	Asn-Ala-Trp-Ala-Val-Gly-His-Leu-Met-NH_2_
[Ala^3^]BN^6–14^	Asn-Gln-Ala-Ala-Val-Gly-His-Leu-Met-NH_2_
BN^6–14^	Asn-Gln-Trp-Ala-Val-Gly-His-Leu-Met-NH_2_
[Ala^5^]BN^6–14^	Asn-Gln-Trp-Ala-Ala-Gly-His-Leu-Met-NH_2_
[Ala^6^]BN^6–14^	Asn-Gln-Trp-Ala-Val-Ala-His-Leu-Met-NH_2_
[Ala^7^]BN^6–14^	Asn-Gln-Trp-Ala-Val-Gly-Ala-Leu-Met-NH_2_
[Ala^8^]BN^6–14^	Asn-Gln-Trp-Ala-Val-Gly-His-Ala-Met-NH_2_
[Ala^9^]BN^6–14^	Asn-Gln-Trp-Ala-Val-Gly-His-Leu-Ala-NH_2_

Seven of the nine peptides studied contain two aromatic amino acids, such as l-tryptophan (Trp) and l-histidine (His). The peptides [Ala^3^]BN^6–14^ (Figure 1C) and [Ala^7^]BN^6–14^ (Figure 2B) contain only His^7^ (at position 7 of the peptide sequence) and Trp^3^, respectively, because the second aromatic amino acid was replaced by Ala. Undoubtedly, the bands at 1587 [ν(ring) + ρ_ipb_(N_1_–H)], 1343 [ν(ring)], 1284 [δ(ring) + ρ_ipb_(C_2_–H)], 1216 (ring berthing), 1181 [δ(ring) + ρ_ipb_(N_1_–H)], and 1153 cm^–1^ [ν(ring)] in the SERS spectrum of [Ala^3^]BN^6–14^ (substitution: Trp^3^ → Ala^3^), adsorbed on the AgNP surface (Figure 1C), are due to in-plane vibrations of the imidazole ring of His^7^.^32,33^ Considering the possible modes of imidazole adsorption at the solution–Ag interface, it can be concluded from the above bands that the free pairs of electrons at the imidazole nitrogen are responsible for the interaction with the surface of AgNPs, causing the imidazole ring to be edge-directed toward the metal surface and remain largely perpendicular to it.^34^ In the SERS spectra of the remaining peptides adsorbed on both metal surfaces, the abovementioned bands are difficult to detect since they overlap with bands due to the indole ring vibrations.

In the SERS spectrum of [Ala^7^]BN^6–14^ (Table 1) adsorbed on the AgNP surface (Figure 2B), the interactions between the indole ring of Trp^3^ and the metal surfaces are indicated by bands at 1608 [W1, benzene + pyrrole and ν(N_1_–C_8_)], 1580 [W2], 1549 [W3, ν(C_2_=C_3_)], 1417 [W6, ν_s_(N_1_C_2_C_3_) + δ(N_1_–H) and benzene δ(CH)], 1359/1341 [W7, indole ν(N_1_–C_8_); Fermi resonance], 1160 [δ(N_1_H)], 1011 [W16 (out-of-phase benzene ring breathing)], 876 [W17, δ(N_1_H) and Fermi resonance], and 758 cm^–1^ [W18, pyrrole ring breathing]. These bands appear in the Raman and SERS spectra of all the peptides studied, except [Ala^3^]BN^6–14^, at similar wavenumbers but show different enhancement. Briefly, in the SERS spectra of [Ala^1^]BN^6–14^, [Ala^2^]BN^6–14^, [Ala^5^]BN^6–14^, and [Ala^8^]BN^6–14^ adsorbed on AgNPs, the W16 and W18 modes (from which the geometry of the indole ring relative to the surface of the substrate can be determined^35^) are stronger than the bands in the corresponding Raman spectra. Although for these peptides deposited on AuNPs, these two SERS signals are very weak compared to their Raman intensity. Considering that bands assigned to other indole modes (e.g., W1, W2, W3, and W7) are observed on the AuNP surface, it can be concluded that the indole ring of [Ala^1^]BN^6–14^, [Ala^2^]BN^6–14^, [Ala^5^]BN^6–14^, and [Ala^8^]BN^6–14^ is parallel to the AuNP surface and perpendicular to the surface of AgNPs.

In the case of the native BN^6–14^ fragment (Table 1), the W16 and W18 SERS signals are very weak in the spectrum on AgNPs (Figure 1D, blue line trace), and W18 is about as intense as its Raman counterpart in the spectrum on AuNPs (Figure 1D, green line trace). This implies that the indole ring is horizontal on AgNPs, whereas on AuNPs, it adopts a tilted orientation toward this surface and interacts with this surface via a pyrrole co-ring. The co-pyrrole ring/AuNP interaction is confirmed by strong 1570 and 1363 cm^–1^ spectral features.

In the case of [Ala^6^]BN^6–14^ and [Ala^7^]BN^6–14^ (Table 1) adsorbed on the surface of the two substrates, the W16 and W18 are weaker than the corresponding Raman bands. Briefly, for [Ala^7^]BN^6–14^ on AgNPs (Figure 2B, blue line trace) and [Ala^6^]BN^6–14^ on AuNPs (Figure 2A, green line trace), the intensity of these bands decreases by about 50 and 90%, respectively, compared to their Raman intensity. For [Ala^7^]BN^6–14^ on AuNPs (Figure 2B, green line trace) and [Ala^6^]BN^6–14^ on AgNPs (Figure 2A, blue line trace), only W18 and W16 are enhanced and show 70 and 50% of Raman intensity, respectively. Thus, the indole ring of [Ala^6^]BN^6–14^ is perpendicular to the AuNP surface, while it is slightly elevated toward the surface of AgNPs, so that the phenyl co-ring faces this surface. On the other hand, the indole ring in [Ala^7^]BN^6–14^ adopts a tilted arrangement on both metal surfaces, with the following difference in the nature of the interaction; both indole co-rings are in contact with AgNPs, while the pyrrole co-ring interacts mainly with AuNPs. This interaction can be supported by the intense 1608, 1549, and 1166 cm^–1^ bands (Figure 2B, green trace line).

For [Ala^9^]BN^6–14^ (Table 1), the W16 and W18 modes in the SERS spectrum on AuNPs (indole ring perpendicular to the substrate surface) increase and decrease in intensity, respectively, for this peptide adsorbed on AgNPs (Figure 2D). The larger intensity loss for W16 compared to W18 may indicate that the indole ring, with the pyrrole edge pointing toward the AgNP surface, adopts a tilted orientation.

In the spectral region from 600 to 850 cm^–1^ of the SERS spectra of all the peptides studied, except [Ala^9^]BN^6–14^, one can expect bands due to the stretching vibrations of the C–S bond [ν(C–S)].^36^ Indeed, these bands are observed for all peptides except for [Ala^8^]BN^6–14^ and [Ala^9^]BN^6–14^, which are adsorbed on both metal surfaces, and [Ala^3^]BN^6–14^, which is immobilized on AgNPs. This means that for these peptides, the Met^9^ side-chain is not involved in adsorption. However, for [Ala^1^]BN^6–14^, [Ala^2^]BN^6–14^, [Ala^4^]BN^6–14^, [Ala^5^]BN^6–14^, and [Ala^6^]BN^6–14^ adsorbed on both metal surfaces, careful analysis of the spectra reveals four bands, whereas for [Ala^7^]BN^6–14^, five overlapping bands can be assigned to the ν(C–S) different conformers of the −CH_2_–CH_2_–S group (Table 2). Of these bands, the SERS signals in the spectra of [Ala^6^]BN^6–14^ on both metal substrates and [Ala^5^]BN^6–14^ on AgNPs are significantly enhanced compared to the normal Raman scattering. The SERS intensities of the ν(C–S) modes for the remaining peptides are stronger than the corresponding Raman intensities and lower than those for [Ala^6^]BN^6–14^ on AgNPs/AuNPs and [Ala^5^]BN^6–14^ on AgNPs.

**Table 2:** Observed Wavenumbers of the ν(C–S) Modes in the SERS Spectra of the Investigated Peptides

		wavenumbers of ν(C–S) [cm^–1^]/conformer					
peptide	substrate	P_H_-G		P_H_-T	P_C_-T	P_C_-G	P_C_-T
[Ala^1^]BN^6–14^	Ag	635		649	677	690	
	Au	607	625	651	670		
[Ala^2^]BN^6–14^	Ag	630		651	674		700
	Au	627		643	670		701
[Ala^3^]BN^6–14^	Ag						
	Au	622		666	681		702
native BN^6–14^	Ag	621		642	688		722
	Au	620	634	663	676		
[Ala^5^]BN^6–14^	Ag	627		658	673	690	
	Au	630		645	663	694	
[Ala^6^]BN^6–14^	Ag	630			672	688	706
	Au	641		653	679	694	
[Ala^7^]BN^6–14^	Ag	633		656	667	700	714
	Au	632		658	675	686	709

The equal number of ν(C–S) bands in the spectra of the given peptide on both surfaces proves that the number of conformations of the Met side-chain does not change, and the high intensity of these bands indicates that the free pair of electrons on the sulfur atom (sp^3^ hybridization) is in direct contact with the metal surface, resulting in the C–S bonds being tilted toward the substrate surface, and as the intensity of these bands decreases, the C–S bond adopts a more perpendicular orientation to the metal surface.

The bands at about 950 [ν(C–C=O)] and 1420 cm^–1^ [δ(CH_2_/CH_3_)] should be assigned to the vibrations of the side-chains of the non-Met residues since these bands are enhanced in the SERS spectra of [Ala^9^]BN^6–14^ (Figure 2D). Taking into account that there is no −COOH group in the structure of the studied peptides, the ∼1397 cm^–1^ SERS signal in the spectra of all peptides on AgNPs and [Ala^1^]BN^6–14^, [Ala^2^]BN^6–14^, [Ala^8^]BN^6–14^, and [Ala^9^]BN^6–14^ on AuNPs is due to the ρ_r_(C=O) + ν(C–N) + δ_as_(CH_3_), ν(C=C/N)_pyrrole_, and/or ν(N_1_C_2_N_3_)_imidazole_ vibrations.^32,37,38^ This suggests that these fragments are involved in peptide adsorption. The intensity of this band is pronounced for [Ala^1^]BN^6–14^, [Ala^2^]BN^6–14^, [Ala^7^]BN^6–14^, and [Ala^9^]BN^6–14^ on AgNPs, indicating direct contact with AgNPs. For these peptides and peptides in whose SERS spectra the ∼1397 cm^–1^ band has a medium intensity (e.g., [Ala^4^]BN^6–14^ on AuNPs and [Ala^5^]BN^6–14^ and [Ala^8^]BN^6–14^ on AgNPs), the SERS signal is significantly enhanced at 950 cm^–1^, confirming the correctness of the proposed assignment and clearly indicating the involvement of the C=O bond in adsorption. Also, the 212–250 cm^–1^ [ν(Ag–O)] and ∼298 and ∼360 cm^–1^ [ν(Au–O)] bands confirm the interaction between the carbonyl group and the metal surface.^39^

The amide I vibration (at about 1670 cm^–1^; disordered structure) shows a medium intensity in the Raman spectrum of BN^6–14^, while it is strongly enhanced in the Raman spectra of other peptides. In the SERS spectrum of [Ala^9^]BN^6–14^ on AuNPs, this band is pronounced, whereas for [Ala^1^]BN^6–14^, [Ala^2^]BN^6–14^, and [Ala^5^]BN^6–14^ on AgNPs and [Ala^8^]BN^6–14^ on both metal surfaces, it shows a medium intensity. It is absent for [Ala^3^]BN^6–14^ on AgNPs and [Ala^5^]BN^6–14^ on AuNPs, but it is observed as a shoulder for other peptides. In contrast to the Raman spectra, in the SERS spectra of all peptides, except [Ala^3^]BN^6–14^ and [Ala^7^]BN^6–14^ on AuNPs, the second amide I band appears at about 1690 cm^–1^ (shoulder), which is associated with a turn structure. Thus, the peptides change their secondary structure upon adsorption.

### SEIRA Spectra Analysis

**Figure 3:**
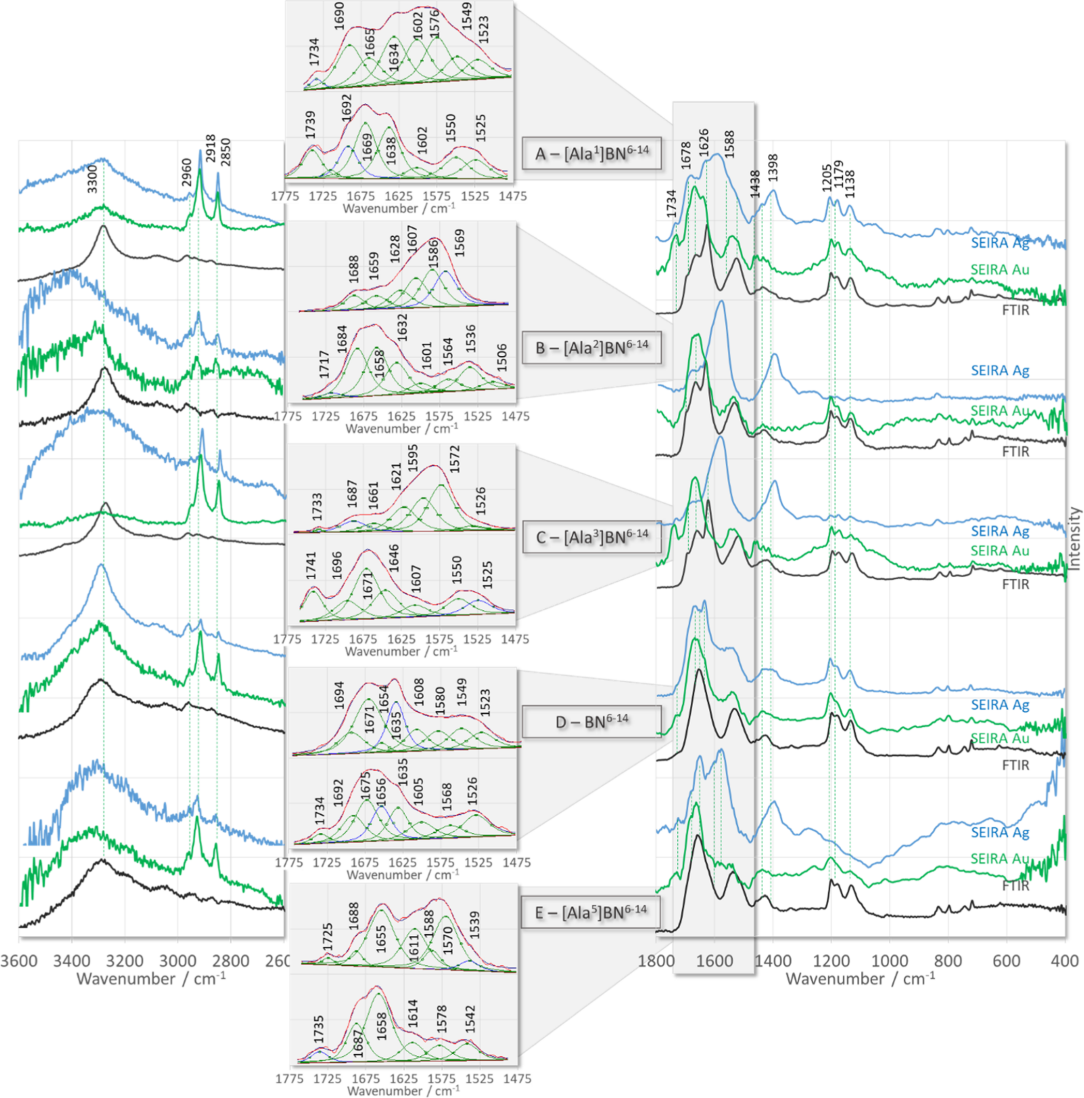
ATR-FTIR (black line traces) and SEIRA spectra of [Ala^X^]BN^6–14^ (where X denotes amino acids at positions 1–5 of BN^6–14^ sequence) adsorbed on the surface of AgNPs (blue line traces) and AuNPs (green line traces).

**Figure 4:**
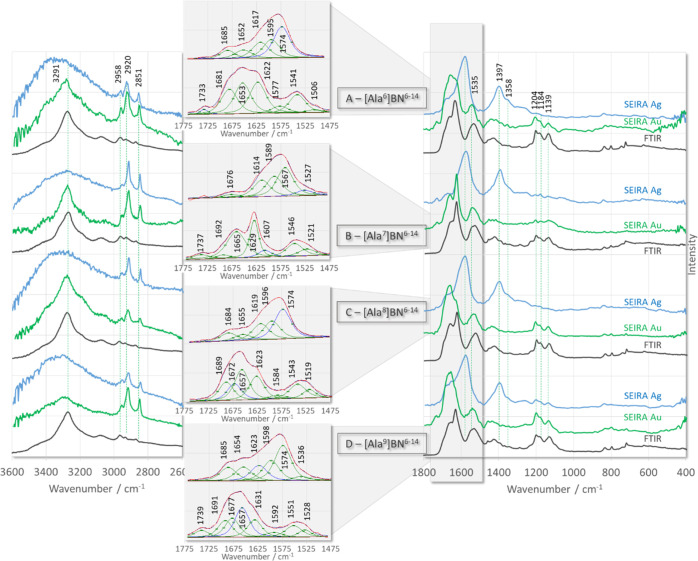
ATR-FTIR (black line traces) and SEIRA spectra of [Ala^X^]BN^6–14^ (where X denotes amino acids at positions 6–9 of BN^6–14^ sequence) adsorbed on the surface of AgNPs (blue line traces) and AuNPs (green line traces).

Figures 3 and 4 show the ATR-FTIR and SEIRA spectra of the studied peptides deposited on the surface of AgNPs and AuNPs together with the results of curve fitting (red line traces) of the SEIRA spectra (black line traces) in the spectral range from 1775 to 1475 cm^–1^, which is advantageous to highlight small relative shifts in the wavenumbers of the bands and allow separation of overlapping bands. The SEIRA spectra in the spectral region below 1250 cm^–1^ are not analyzed in detail. Instead, Table 3 summarizes the observed bands and their assignments based on vibrational spectroscopy results since there are few literature reports available on SEIRA studies of peptides. This is because many research groups have focused on the preparation of new substrates and the determination of signal enhancement and SEIRA mechanism using adsorbates,^40^ which usually contain carbonyl and thiol groups^5^ or adsorbates in the form of small and/or symmetric molecules^41−45^ and less frequently thin polymer films.^46−48^ Few literature reports also indicate the use of SEIRA in the study of, for example, biosensors of antigen–antibody (Salmonella) interactions,^40^ membrane proteins,^49−53^ albumin, nucleobases, and nucleic acids,^54^ tobacco mosaic virus,^55^ and cancer drug research (*cis*-platinum and doxorubicin)^56^ and as a diagnostic criterion for cancer.^57^

**Table 3:** Assignment of the SEIRA Bands in the Spectral Region below 1250 cm^–1^^a^

assignment^31^	cm^–1^
δ(CCH_2_S/C), ρ_b_(C(H,N_A_)C/C_A_), ρ_b_(C–NH_2_)	1204
ρ_r_(CH_2_), ν(C–C)	1183
ν_as_(CCN), δ(C–NH_2_), δ(NCH_2_C_A_), ν(C-N_A_), δ(CCH_2_C)	1139
His [ρ_ipb_(C–H)], ρ_τ_(NH_2_)	1098
ν(C–C)	1018
ρ_r_(CH_2_), δ(CCH_2_S), δ(S–CH_3_), ρ_oop_(C–NH_2_), δ(CC_A_O_A_N_A_)	890
ν(CNC)_secondary amide_	860
ν_as_(CSC)	835
ρ_r_(CH_2_), ν(C–S)	780
ν(C–S)	743
ν(C–S)	724
ν(C–S)	621
δ(CC=O), ρ_oop_(C_A_N_A_HC), γ(CC_A_O_A_N_A_HC)	538
δ(CC=O), ρ_τ_(NH_2_)	508
ρ_τ_(NH_2_)	444

a Abbreviations: ν—stretching, ν_as_—asymmetric stretching, ρ_b_—bending, ρ_ipb_—in-plane bending, ρ_r_—rocking, ρ_τ_—twisting, δ—deformation, and δ_oop_—out-of-plane deformation vibrations, and A—amide bond atom.

Since the thiol group (−CSH) adsorbs radiation very poorly in the IR range (low dipole moment of the C–S and S–H bonds), it produces very weak bands in the infrared spectra. In contrast, the dipole moment of polar bonds such as O–H, C=O, and N–H (found in amide bonds and functional groups) changes the most during the vibrations of the molecule, and therefore, they produce strong bands in the infrared spectra, most of which can be observed in the spectral region above 1500 cm^–1^ of the SEIRA spectra. Structural information can be obtained by analyzing the amide bands (−CONH−) in the SEIRA spectra, particularly amide I and II (of relatively strong infrared intensity), whose wavenumbers are sensitive to peptide chain conformation (e.g., α-helices, β-sheets, turns, and disordered structure) and hydrogen bonding in the peptide backbone.

As shown in Figures 3 and 4, the width of the contributing component bands within the amide I and II regions (above 1500 cm^–1^) is greater than the distance between the maxima of adjacent bands. As a result, the individual component bands cannot be separated in the experimental spectra. Using the curve-fitting procedure, we were able to increase the separation of the overlapping components present in the broadband envelope and reveal many components. With additional consideration of selective substitution at positions 3 (Trp^3^ → Ala^3^) and 7 (His^7^ → Ala^7^) for peptides [Ala^3^]BN^6–14^ (Figure 3C) and [Ala^7^]BN^6–14^ (Figure 4B), respectively, the fitted bands were assigned to normal vibrations and are summarized in Table 4. On the surface of AgNPs, in general, the fitted bands at higher wavenumbers (1775–1610 cm^–1^) are stronger than those at lower wavenumbers (1610–1475 cm^–1^), except for [Ala^4^]BN^6–14^ (Figure 3D), where the bands at higher frequencies are less intense than those at lower frequencies. On the surface of AuNPs, the band intensities are inverted, that is, the bands in the lower wavenumber region give a less intense band envelope than the overlapping bands on the higher frequency side, except for [Ala^1^]BN^6–14^ (Figure 3A) and [Ala^5^]BN^6–14^ (Figure 3E), where the band envelope intensity is comparable in both regions.

**Table 4:** Assignment of the Curve-Fitted SEIRA Bands in the Spectral Region from 1775 to 1475 cm^–1^

	[Ala^1^]BN^6–14^		[Ala^2^]BN^6–14^		[Ala^3^]BN^6–14^		BN^6–14^		[Ala^5^]BN^6–14^		[Ala^6^]BN^6–14^		[Ala^7^]BN^6–14^		[Ala^8^]BN^6–14^		[Ala^9^]BN^6–14^	
	Asn^1^ → Ala^1^		Gln^2^ → Ala^2^		Trp^3^ → Ala^3^		Ala^4^		Val^5^ → Ala^5^		Gly^6^ → Ala^6^		His^7^ → Ala^7^		Leu^8^ → Ala^8^		Met^9^ → Ala^9^	
assignment	Ag	Au	Ag	Au	Ag	Au	Ag	Au	Ag	Au	Ag	Au	Ag	Au	Ag	Au	Ag	Au
ν(C=O)	1734	1739		1717	1733	1741		1734	1725	1735		1733		1737				1739
amide I [ν(C=O) + ν(N −C)] (turn)	1690	1692	1688	1684	1687	1696	1694	1692	1688	1687	1685	1681		1692	1684	1689	1685	1691
amide I [ν(C=O) + ν(N −C)] (disordered)	1665	1669			1661	1671	1671	1675					1676	1665		1672		1677
His [ν(C_4_=C_5_)], δ_sym_(NH_3_^+^)			1659	1658		1646	1654	1656	1655	1658	1652	1653			1655	1657	1654	1657
ν(C=C)	1634	1638	1628	1632	1621		1635	1635	1611	1614	1617	1622	1614	1629	1619	1623	1623	1631
Trp [W1, phenyl + ν(C_8_–N_1_)], His [ν(ring)]	1602	1602	1586	1601	1595	1607	1608	1605	1588		1595	1577	1589	1607	1596	1584	1598	1592
His [ν(ring) + ρ_ipb_(N_1_H)],Trp [W2, phenyl]	1576		1569	1564	1572		1580	1568	1570	1578	1574		1567		1574		1574	
Trp [W3, ν(C_2_=C_3_)], ρ_b_(NH_2_), δ(C–NH_2_)	1549	1550		1536		1550	1549		1539	1542		1541		1546		1543	1536	1551
amide II [ν(N–C) + ρ_ipb_(NH)]	1523	1525			1526	1525	1523	1526					1527	1521		1519		1528
His [ν(ring) + ρipb(C_2_H)]				1506								1506						

The absence of the ν(C=O) band for [Ala^8^]BN^6–14^ (Figure 4C) on AgNPs (blue line trace) and AuNPs (green line trace) indicates that the >C=O unit is not located near the surface of the two substrates. The said-chain of Asn^1^ and Gln^2^, the amidated *C*-terminus of the peptides, and the peptide bonds consist of the >C=O unit. Peptide bonds for all peptides, except [Ala^7^]BN^6–14^ on AgNPs, give the amide I band at 1696–1981 cm^–1^ (turn structure). While for [Ala^1^]BN^6–14^, [Ala^3^]BN^6–14^, [Ala^4^]BN^6–14^, and [Ala^7^]BN^6–14^ absorbed on the surface of both substrates, as well as for [Ala^8^]BN^6–14^ and [Ala^9^]BN^6–14^ deposited on AuNPs, both the amide I (1677–1661 cm^–1^) and amide II (1521–1528 cm^–1^) bands of the disordered structure were calculated. These bands indicate a conformational change when the peptide interacts with the metal surface, that is, the formation of a turn structure contacting the metal surface. Similar conclusions were drawn from biological activity studies, that is, bombesin was shown to interact with GPCRs via a turn structure located at the *C*-terminus of the peptide. The stronger enhancement of the amide I mode on the AuNP surface compared to the AgNP surface for [Ala^2^]BN^6–14^, [Ala^3^]BN^6–14^, [Ala^6^]BN^6–14^, [Ala^7^]BN^6–14^, and [Ala^8^]BN^6–14^ further indicates the greater strength of the interaction between the peptide bond and the AuNPs, which is probably related to the fact that the free pair of electrons on the oxygen atom (sp^2^ hybridization) of the peptide bond has easier access to this surface, that is, the C=O bond is inclined to the AuNP surface (more or less than 120°), whereas on the AgNP surface, the bond is more perpendicular to this surface.

ν(C=O) appears in the spectra of all peptides on AuNPs and only in the spectra of [Ala^1^]BN^6–14^, [Ala^3^]BN^6–14^, and [Ala^5^]BN^6–14^ on AgNPs. This observation suggests that the ∼1737 cm^–1^ SERS signal is related to the C=O bond vibrations in the side-chain of Asn^1^ and Gln^2^, and the spectral features at ∼1398/1620 cm^–1^ are due to the vibrations of the *C*-terminal group. Therefore, the ρ_b_(NH_2_) and δ(C–NH_2_) modes are expected to be present in the spectra of those peptides for which ν(C=O) is observed, and conversely, the ρ_b_(NH_2_) + δ(C–NH_2_) modes will not be present in the peptide spectra for which ν(C=O) is not enhanced. This is the case. For example, in the SEIRA spectra of [Ala^1^]BN^6–14^ and [Ala^5^]BN^6–14^ immobilized on the surface of the two substrates, and [Ala^2^]BN^6–14^, [Ala^3^]BN^6–14^, [Ala^6^]BN^6–14^, [Ala^7^]BN^6–14^, [Ala^1^]BN^6–14^, and [Ala^9^]BN^6–14^ adsorbed on AuNPs, both these modes are present, whereas for [Ala^2^]BN^6–14^, [Ala^6^]BN^6–14^, [Ala^7^]BN^6–14^, and [Ala^8^]BN^6–14^ on AgNPs, neither mode occurs. For the remaining peptides, only one of these bands is calculated. This effect is probably related to the weak C=O···metal interaction (low band intensity at 1733 cm^–1^), leading to weak or no enhancement of ρ_b_(NH_2_)/δ(C–NH_2_) (for [Ala^3^]BN^6–14^ on AgNPs and [Ala^4^]BN^6–14^ on AuNPs) or the fact that the ∼1550 cm^–1^ SERS signal is due to the aromatic ring vibrations of Trp^3^ (for [Ala^4^]BN^6–14^ and [Ala^9^]BN^6–14^ on AgNPs and [Ala^8^]BN^6–14^ on AuNPs).

The bands at 1595–1607 and 1567–1572 cm^–1^ in the SERS spectra of [Ala^3^]BN^6–14^ and [Ala^7^]BN^6–14^ can undoubtedly be attributed to the aromatic ring vibrations of His^7^ and Trp^3^, respectively. Unfortunately, since the bands of these two aromatic rings occur in the same spectral regions, it is difficult to assign a particular band to the vibrations of a particular ring in the spectra of the other peptides. However, assuming that the weak band at 1506 cm^–1^ corresponds to the imidazole vibrations, it can be proposed that His^7^ for [Ala^2^]BN^6–14^ and [Ala^6^]BN^6–14^ is involved in the interaction of these peptides with the AuNP surface.

### Biological Activity Studies

**Figure 5:**
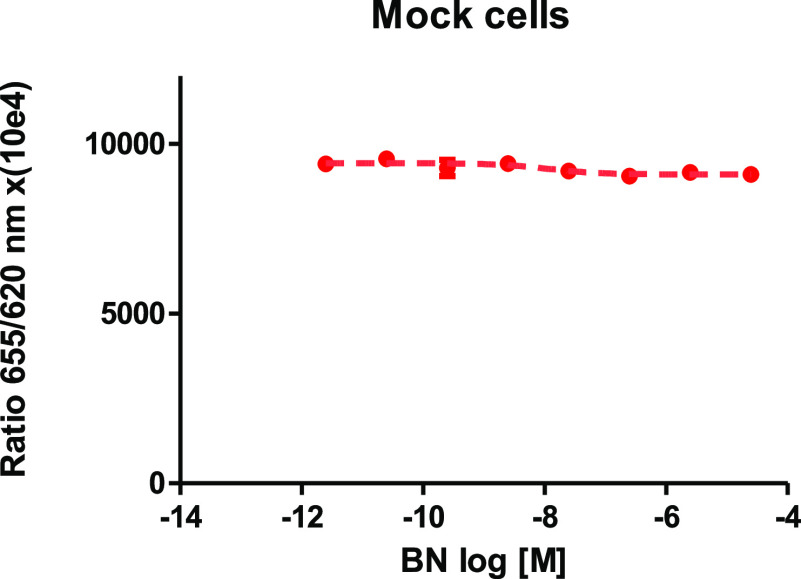
Influence of BN on IP1 level in the mock HEK-293 cell line. This graph demonstrates that no unspecific activity was caused by BN, and there is no expression of any bombesin receptor.

First, the concentration of IP1 was measured in mock cells without transfection of any bombesin receptors. As presented in Figure 5, the HEK-293 cell line did not elevate the intracellular IP1 concentration in the presence of a different concentration of BN.

**Figure 6:**
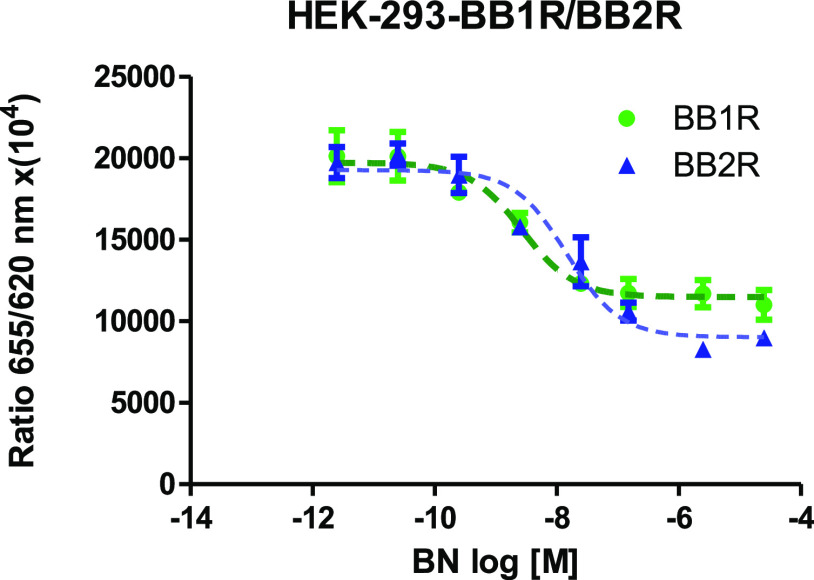
Effect of BN on HEK-293 cells transfected with cDNA for human BB1 or BB2 receptor. Dose-dependent elevation of the intracellular level of IP1 in the presence of BN was observed. The signal generated by this IP-One (Cis-Bio) is inversely proportional to the amount of IP1.

Proper bombesin receptors 1 or 2 expression and function were confirmed in functional IP1 assay. The agonist BN elevated the intracellular IP1 concentration in a dose-dependent manner. EC_50_ was estimated for BB1R 14.6 nM and BB2R 2.6 nM (Figure 6).

**Figure 7:**
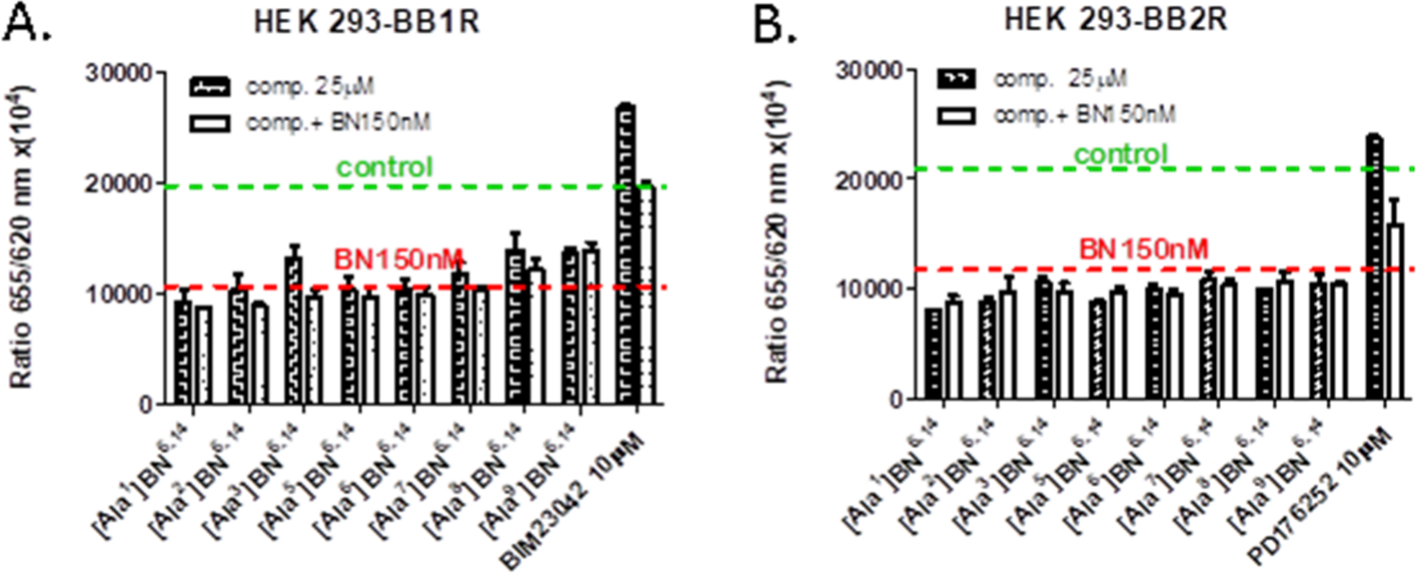
Effect of alanine-scanning of the bombesin fragment on biological activity. Analogues of bombesin with Ala substitution were administered to cells with BB1R (A) or with BB2R (B) expression alone or coadministered with BN 150 nM. The green dashed line corresponding to the IP1 level for control cells without BN, and the red dashed line is the IP1 level for bombesin in a concentration of 150 nM.

Next, the biological activity of bombesin derivatives was analyzed. The cells were incubated in compounds alone (25 μM) or in the presence of bombesin 150 nM to check the potential inhibitory effect as antagonists. Additionally, reference compounds BIM23042 and PD176252 (both from Tocris) antagonists of BB1 and BB2 receptors were used in a concentration of 10 μM (Figure 7).

As presented in Figure 7, none of the derivatives showed antagonist properties. All compounds alone induced receptor activation and elevation of intracellular IP1 concentration and did not reverse the agonist effect. In contrast, reference antagonists (BIM23042 and PD176252) inhibited the basal activity of both receptors and in the presence of agonist downregulated IP1 concentration to control level (cells only) (Figure 7A) or significantly reduced its level (Figure 7B).

Our data showed that the selective substitution of an amino acid in the 6–14 *C*-terminal fragment of bombesin by alanine did not preclude its agonistic activity, in contrast to the results described by Horwell et al.^58^ They showed that the alanine scan of Ac-BN^7–14^ reduced the biological activity at few alanine substitutions and changed the Ki from the nanomolar to the micromolar scale. The discrepancies between our and Horwell et al. studies may arise from the use of different experimental procedures. First, a slight difference in the length of the peptide between eight and nine amino acids is observed. Also, the *N*-terminus in the 8aa peptide was modified by acetylation.^58^ The second difference may be due to different biological models used in the functional assays. In our work, we used human HEK-293 cells to relate the obtained results to the human body. In the scientific literature, we find information about the differences in functional studies due to the use of different types of host cells.^59,60^ The differences between the activity of GPCRs between cell lines involve the level and composition of the G protein pool expressed in the host cells, post-transcriptional modification of GPCRs such as differences in splice variants, and modification of the amino acid sequence by adding different types of molecules or chemical groups.^59,61,62^

## Conclusions

Many tumors synthesize bombesin (BN), an important neurotransmitter known as an autocrine tumor growth factor, and overexpress GPCRs of this peptide. Therefore, the BN-GPCR system is being investigated for use in novel anti-cancer therapies, using overexpressed receptors for imaging and targeting cytotoxic compounds, either by direct conjugation or in combination with metal nanoparticles. Therefore, it is important to understand the adsorption of this peptide on the surface of metal NPs. Information on adsorption can be obtained by SERS and SEIRA. The effect of a particular amino acid in a peptide sequence on adsorption can be determined by selective mutagenesis, known as alanine-scanning.

In this work, native and eight single Ala-substituted 6–14 *C*-terminal fragments of BN, [Ala^X^]BN^6–14^ (where X takes values from 1 to 9 and represents a position in the BN^6–14^ sequence) were synthesized. These substitutions allowed us to answer the questions: (1) whether the adsorption changes due to amino acid substitution at a particular position of the peptide chain and how it changes on AgNP and AuNP surfaces and (2) how the biological activity of the peptide changes as a consequence of a single amino acid substitution in the peptide sequence. The use of 6–14 *C*-terminal fragments of BN instead of the native BN was dictated by the fact that studies to determine the peptide-binding domain and receptor activation requirements, using mutant bombesin analogues obtained by trial and error, showed that the nano-peptide (BN^6–14^) is a fragment that interacts with GPCRs or induces biological activity like the full-length BN.^63−65^

The [Ala^X^]BN^6–14^ peptides were immobilized on the surface of AgNPs and AuNPs, which are readily available and have a uniform shape and diameter, which may bring us closer to the more common and routine use of SERS/SEIRA. Complex SEIRA spectra were analyzed using the curve-fitting procedure, which had the advantage of highlighting small relative shifts in the wavenumbers of the bands and allowing separation of overlapping bands.

Evidence was provided to confirm the validity of SERS/SEIRA to select those peptide fragments within the studied group of peptides that play a role in substrate–receptor interactions in systems where biological studies are difficult or do not lead to clear determination of the peptide fragments responsible for biological activity. The SERS/SEIRA and biological activity results showed:

1 The peptide structure changes from irregular to turn type due to the binding of the peptide to the metallic surface. The peptides [Ala^1^]BN^6–14^, [Ala^3^]BN^6–14^, and [Ala^7^]BN^6–14^ behave like a native fragment of BN ([Ala^4^]BN^6–14^), which means that the modification at positions 1, 3, and 7 does not affect the conformational change. The biological studies reported in the literature also indicate that the active conformation of BN when interacting with GPCRs is a turn structure at positions 10–13 and hydrogen bonds between the amide NH_2_ of l-methionine at position 14 of the amino acid sequence (Met^14^) of BN and C=O of Trp^8^, C=O of Leu^13,^ and N–H of Val^10^, and between N–H of Leu^13^ and C=O of Val^10^.^66−69^
2 The number of Met side-chain conformations (four) does not change upon selective substitution, except for [Ala^8^]BN^6–14^ and [Ala^9^]BN^6–14^ (no interaction) and [Ala^7^]BN^6–14^ (five rotamers) on two substrates and [Ala^3^]BN^6–14^ on AgNPs (no interaction). Similar to [Ala^4^]BN^6–14^, the *C*-terminal group of all peptides is involved in the peptide interaction with two surfaces (strongly with AgNPs and weakly with AuNPs). Previous results showed that the *C*-terminal Met side-chain is not essential for BN agonistic activity since other diverse amino acid substitutions in this position, for example, norleucine, also yield agonists.^69−71^ However, it has been shown that the carboxamide group at this position is crucial for BN expression of biological activity as its removal always yields pure antagonists.^69,72^
3 The side-chain of Asn^1^ or Gln^2^ of the native peptide interacts with the AuNP surface, as is the case for the peptides [Ala^2^]BN^6–14^, [Ala^6^]BN^6–14^, [Ala^7^]BN^6–14^, and [Ala^9^]BN.^6–14^ This means that the modification at positions 2, 3, 6, 7, and 9 does not prevent the C=O···AuNP interaction. Previous results showed that the C=O unit of Gln^7^ plays a key role in receptor pathway recognition in mammalian pancreatic-acinar cells.^71^
4 Trp of all peptides, except [Ala^3^]BN^6–14^, and His of [Ala^2^]BN^6–14^ and [Ala^6^]BN^6–14^ on AuNPs interact with surface substrates. Previous results showed that deletion or substitution of Trp^8^ generates an inactive analogue. Therefore, Trp^8^ is thought to be responsible for receptor recognition.^73^ However, recent studies show that Trp^8^ substitution produces little change in the activity of [Ala^3^]BN^6–14^.
5 The biological activity studies using HEK-293 cells as an excellent model for drug screening showed for the first time that the newly synthesized bombesin derivatives exhibit agonistic properties.

## Experimental Section

### Peptides

Peptides were synthesized via the solid-phase method using the Fmoc/*t*Bu strategy, as previously described^74^ with minor modifications. Briefly, TentaGel S RAM resin (Rapp Polymere, Germany) was used. The elongation of the peptide chain was performed using an automatic peptide synthesizer (Symphony, Gyros Protein Technologies, USA) in the reaction with a threefold excess of respective Fmoc-amino acid in an equimolar mixture with *O*-(7-azabenzotriazol-1-yl)-*N*,*N*,*N′*,*N′*-tetramethyluronium hexafluorophosphate (HATU) and 1-hydroxy-7-azabenzotriazole (HOAt) and two equivalents of *N*-methylmorpholine (NMM). Fmoc-protected amino acids were purchased from commercial supplies (Merck KGaA, Germany and GL Biochem, China). Peptide cleavage was performed using a standard mixture in a 2 h reaction. Crude peptides were precipitated with cold diethyl ether, centrifuged, dried, dissolved in water, and lyophilized overnight.

Peptides were purified by a preparative reversed-phase high-performance liquid chromatography (RP-HPLC) system (Waters, USA) equipped with a Jupiter Proteo column (4 μm, 90 Å, 250 × 10 mm). The purity (purity is >95%) of peptides was determined using an analytical RP-HPLC system (Shimadzu, Japan) with a Jupiter Proteo column (4 μm, 90 Å; 250 × 4.6 mm) and the linear gradient of solution B in A from 1 to 80% in 30 min with a flow rate of 1 mL/min. Used eluents were A—0.1% aqueous solution of TFA and B—80% solution of acetonitrile in aqueous 0.1% TFA (v/v). The mass spectra of peptides were recorded on a Bruker BIFLEX III MALDI TOF mass spectrometer (see the Supporting Information, analytical data of synthesized peptides).

### Colloids

Three batches of gold [Au nanospheres (AuNPs) with a diameter of 20 nm, ∼7.2 × 10^10^ particles/mL, stabilized suspension in 0.1 mM phosphonate-buffered saline, reactant free, and polydispersity index <0.2, λ_max_ = 529–533 nm] and silver [Ag nanospheres (AgNPs) with a diameter of 40 nm, 0.02 mg/mL in water, polyvinylpyrrolidone functionalized, λ_max_ = 415 nm] colloidal solutions were obtained from Merck (Poland).

### ATR-FTIR and SEIRA Measurements

Prior to SEIRA measurements, each peptide (30 μL of an aqueous peptide solution) was immobilized on colloidal suspension (10 μL). The peptide/colloidal nanoparticle mixture was then loaded onto a diamond ATR adapter and allowed to dry. Unbound peptide molecules were removed by washing with deionized water and allowed to dry. The procedure was repeated three times.

Spectra were recorded using an FTIR spectrometer Thermo Scientific Nicolet 6700 equipped with a diamond ATR accessory. Measurement conditions: a resolution of 4 cm^–1^ and 128 scans.

### Raman and SERS Measurements

Aqueous solutions of each peptide were prepared at a concentration of 10^–4^ mol/L and a pH of 7. 10 μL of the peptide solution was deposited onto three Au or Ag surfaces. The SERS spectra were collected three times at three different locations on each surface.

Nine samples of 40 μL nanoparticle solutions (three samples from three different batches of Ag or Au colloid) were mixed with 20 μL of the each peptide solution. The final sample concentration was 3 × 10^–5^ mol/L (no conventional Raman signal was observed at this sample concentration). The 20 μL peptide/sol mixture was applied to the glass plate, and the SERS were recorded (no measurements were made for the dried droplet).

Raman and SERS spectra were recorded using an inVia Raman spectrometer (Renishaw) containing an air-cooled charge-coupled device detector and a Leica microscope (50× objective). The spectral resolution was set to 4 cm^–1^. The 785 nm line of a diode laser was used as the excitation source. The laser power at the output was set at 20 mW. The typical exposure time for each Raman and SERS measurement was 40 s with five accumulations (series of five spectra, each accumulated 40 s = 200 s). The SERS spectra of a given peptide adsorbed on AgNPs or AuNPs from three different batches (bottles) were almost identical, except for small differences (up to 5%) in some band intensities. During the measurements, no spectral changes were observed that could be related to the sample decomposition.

### Spectral Analysis

Spectral analysis was performed using the software package GRAMS/AI 8.0 (Thermo Electron).

Deconvolution of the 1775–1475 cm^–1^ spectral region for the SERS spectra of the investigated peptides was conducted adopting a 50/50% Lorentzian/Gaussian band shape. The number of bands was selected based on a careful analysis of the spectra and their second-derivative spectra.

### Cell Culture and Transfection

Cell lines overexpressing human bombesin receptors 1 and 2 were prepared by transfection of HEK-293 cell line. The human BB1 receptor NM_002511.3 (GenScript) and BB2 receptor NM_002091 (cDNA Resource Center) sequences in pcDNA 3.1 + plasmids were introduced into the host cell by lipofection (Lipofectamine Reagent 3000, Thermo Fisher Scientific). Then, the transfected cells were selected with G-418 antibiotic at a dose of 500 μg/mL. Cells were grown under standard cell culture conditions (37 °C, 5% CO_2_) in DMEM supplemented with 10% FBS and Glutamax.

### Intracellular Inositol Monophosphate (IP-One) Assay

Receptor activity was determined by measuring the intracellular inositol monophosphate concentration using a homogeneous time-resolved fluorescence IP-one kit from Cis-bio, according to the manual. Briefly, 24 h before assay, cells were grown in an FBS-free DMEM medium. Then, the cells were scraped and centrifuged. The cell pellet was suspended in Hanks-HEPES (130 mM NaCl, 5.4 mM KCl, 1.8 mM CaCl_2_, 0.8 mM MgSO_4_, 0.9 mM NaH_2_PO_4_, 20 mM HEPES, LiCl 50 mM, and 3.25 mM glucose; pH 7.4). Then, the cell suspension was incubated for 1 h (37 °C) on a 384 white low-volume plate (Grainer Bio-One) in the presence of an increased concentration of bombesin or a 25 μM concentration of bombesin analogues alone or in the presence of a bombesin concentration corresponding to EC_80_ (total volume, 10 μL; 2 × 10^4^ cells). The cell suspension was then mixed with 5 μL of the IP1-acceptor conjugate and 5 μL of the anti-IP1-donor conjugate. After 1 h incubation at RT, fluorescence was read at 620 and 665 nm (Tecan; Infinite M1000). Results were calculated as the ratio (665 nm/620 nm) multiplied by 10^4^. The signal was inversely proportional to the concentration of IP1 in the samples. Each sample was prepared in triplicate. Data were analyzed using GraphPad Prism version 5.04 for Windows (GraphPad software).

## Supporting Information Available

Analytical data of synthesized peptides (PDF)
Molecular formula strings (CSV)

## Abbreviations

AgNPs: silver nanoparticles
ATR-FTIR: attenuated total reflection-Fourier transform infrared spectroscopy
AuNPs: gold nanoparticles
BN: bombesin
GPCRs: membrane-bound G protein-coupled receptors
RS: Raman spectroscopy
SEIRA: surface-enhanced infrared spectroscopy
SERS: surface-enhanced Raman spectroscopy
