# Supplementary material for: Application of Alanine Scanning to Determination of Amino Acids Essential for Peptide Adsorption at the Solid/Solution Interface and Binding to the Receptor: Surface-Enhanced Raman/Infrared Spectroscopy versus Bioactivity Assays
Source: J Med Chem. 2021 Jun 10;64(12):8410–22. doi: 10.1021/acs.jmedchem.1c00397 (PMC8279479; doi:10.1021/acs.jmedchem.1c00397)

Supporting Information

Application of alanine-scanning to determination amino acids essential for peptide adsorption at the solid/solution interface and binding to the receptor: surface-enhanced Raman/infrared spectroscopy vs. bioactivity assays

Edyta Proniewicz[a]*, *Grzegorz Burnat*[b], *Helena Domin*[b], *Izabela Małuch*[c], *Marta Makowska*[c], *Adam Prahl*[c]

[a]Faculty of Foundry Engineering, AGH University of Science and Technology, 30-059 Krakow, Poland

[b]Maj Institute of Pharmacology, Polish Academy of Sciences, Department of Neurobiology, 12 Smętna 12, 31-343 Kraków, Poland

[c]Faculty of Chemistry, University of Gdansk, Wita Stwosza 63, 80-308, Gdansk, Poland

*corresponding author: proniewi@agh.edu.pl

Analytical data of synthesized peptides

pGlu-Gln-Arg-Leu-Gly-Asn-Gln-Trp-Ala-Val-Gly-His-Leu-Met-NH2, BN

$[M+H]^+$ calculated 1619.871, found 1619.666

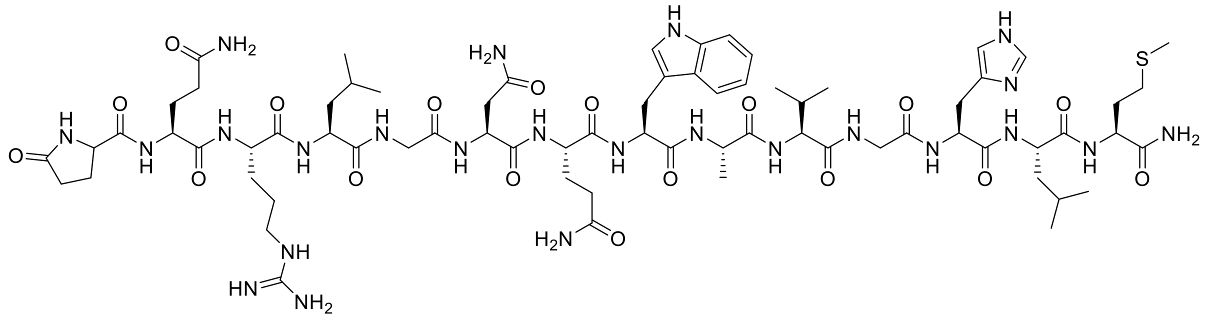

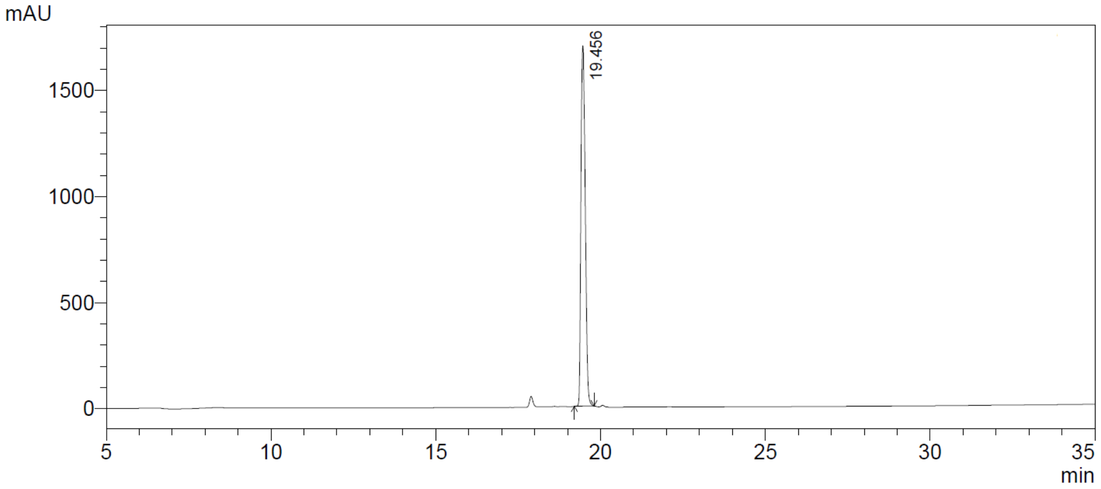

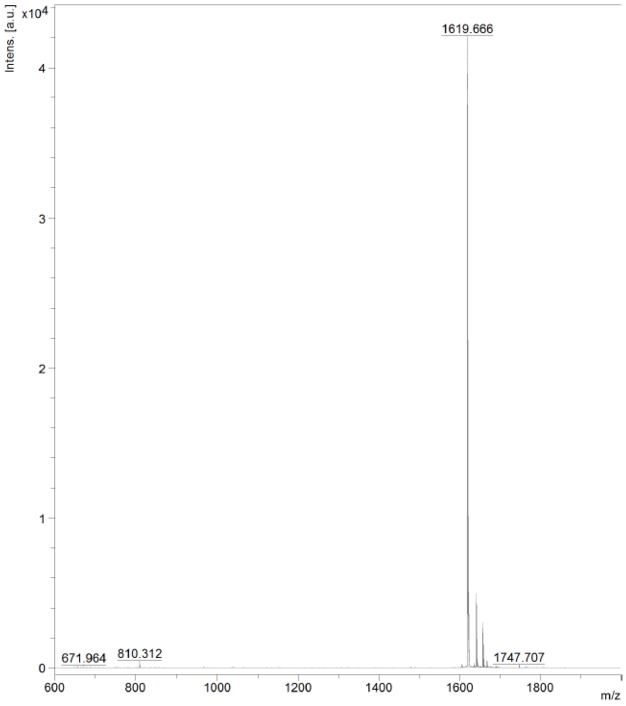

Ala-Gln-Trp-Ala-Val-Gly-His-Leu-Met-NH2, **[Ala1]BN6-14**

$[M+H]^+$ calculated 1011.214, found 1011.431

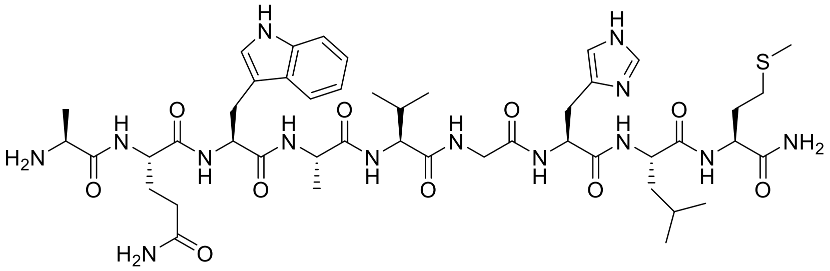

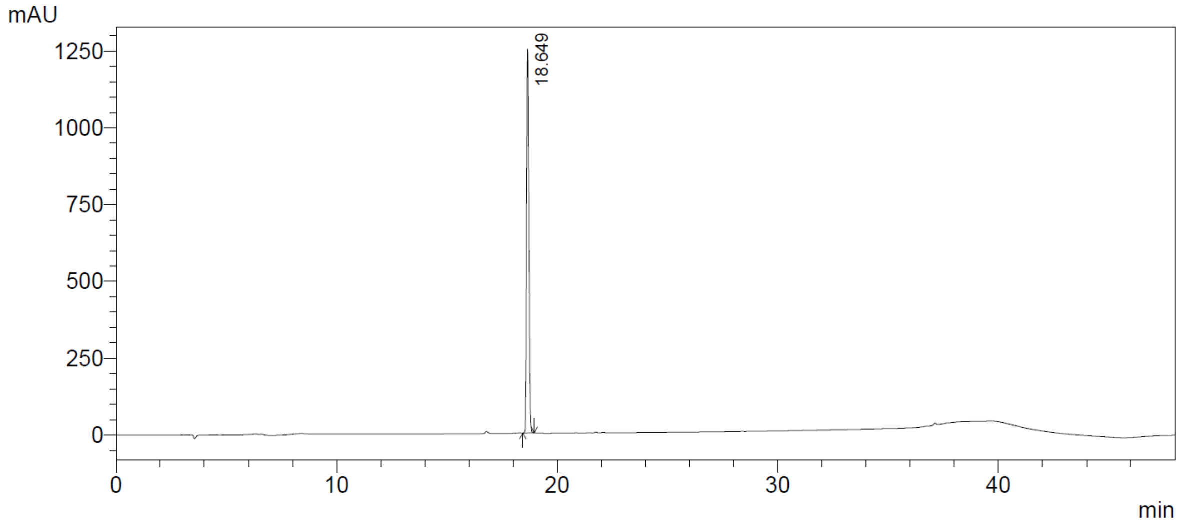

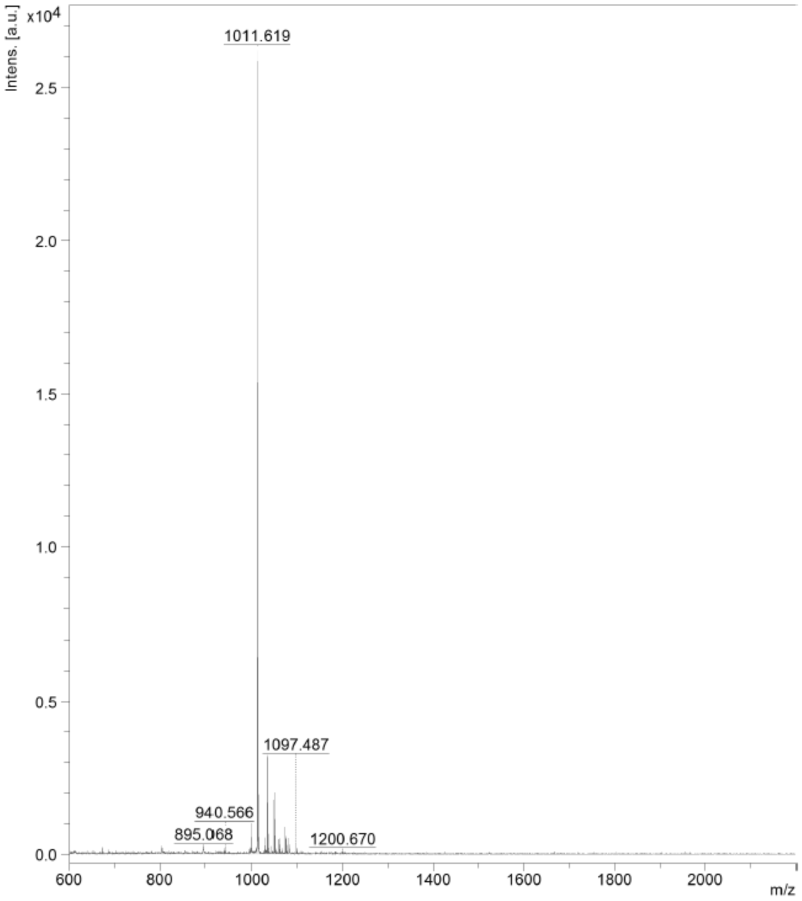

Asn-Ala-Trp-Ala-Val-Gly-His-Leu-Met-NH2 , [Ala²]BN⁶⁻¹⁴

$[M+H]^+$ calculated 997.187, found 997.445

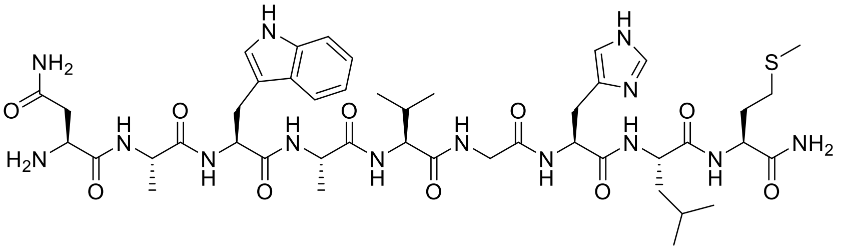

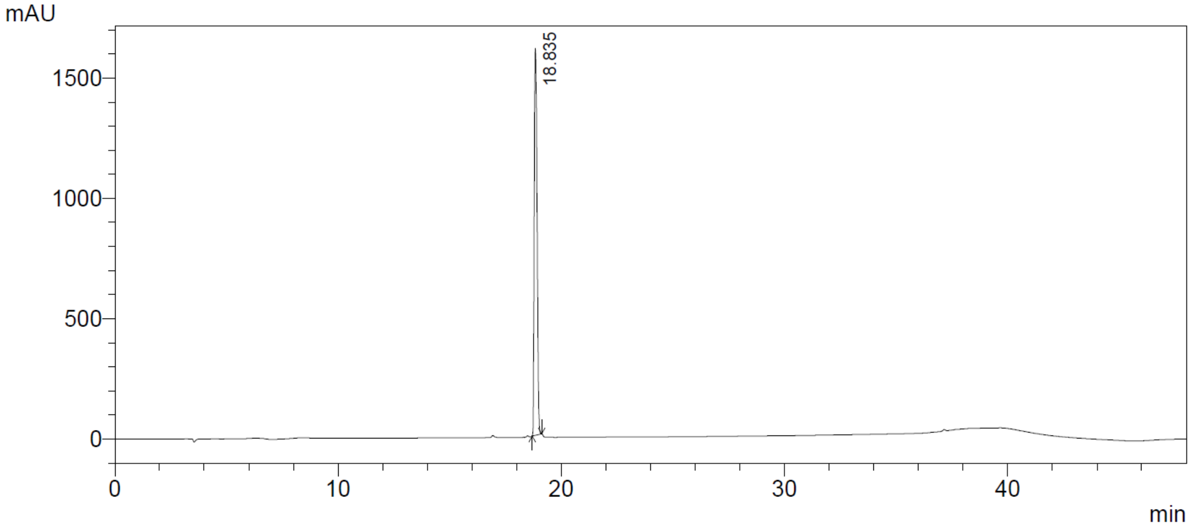

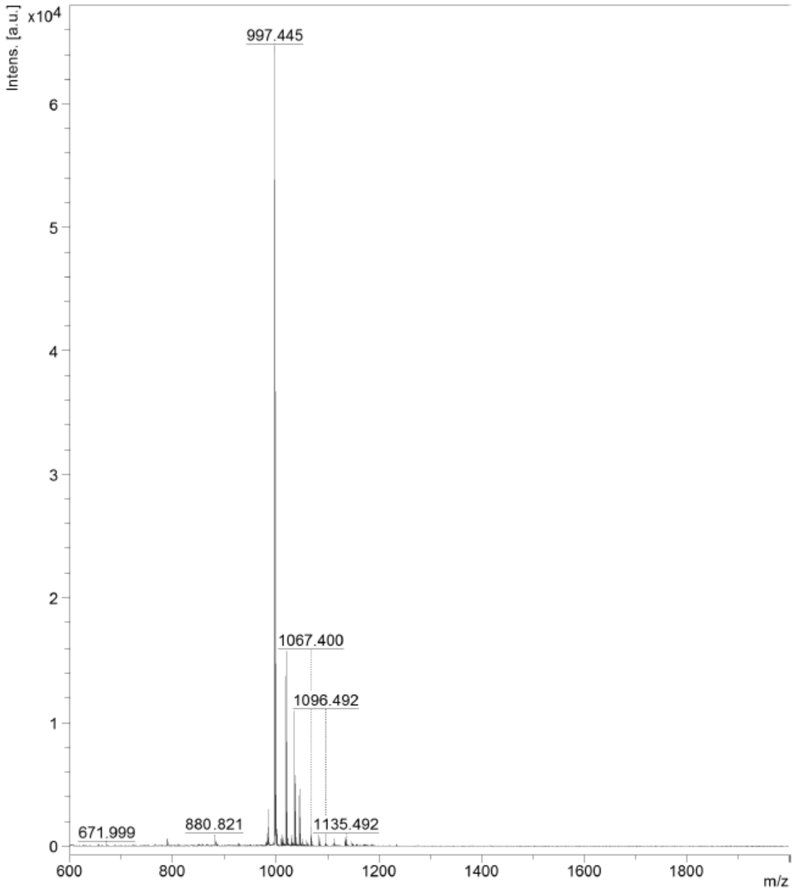

Asn-Gln-Ala-Ala-Val-Gly-His-Leu-Met-NH2 ,   [Ala[3]]BN[6-14]

$[M+H]^+$ calculated 939.104, found 939.420

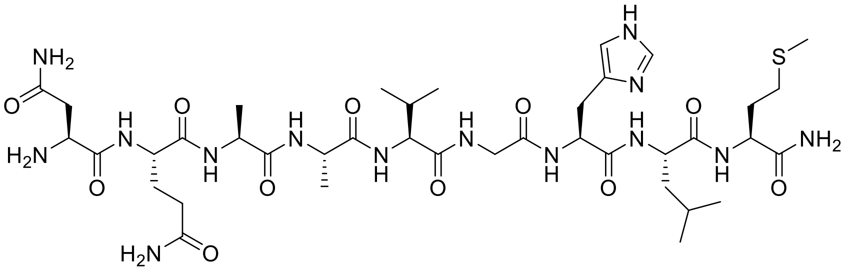

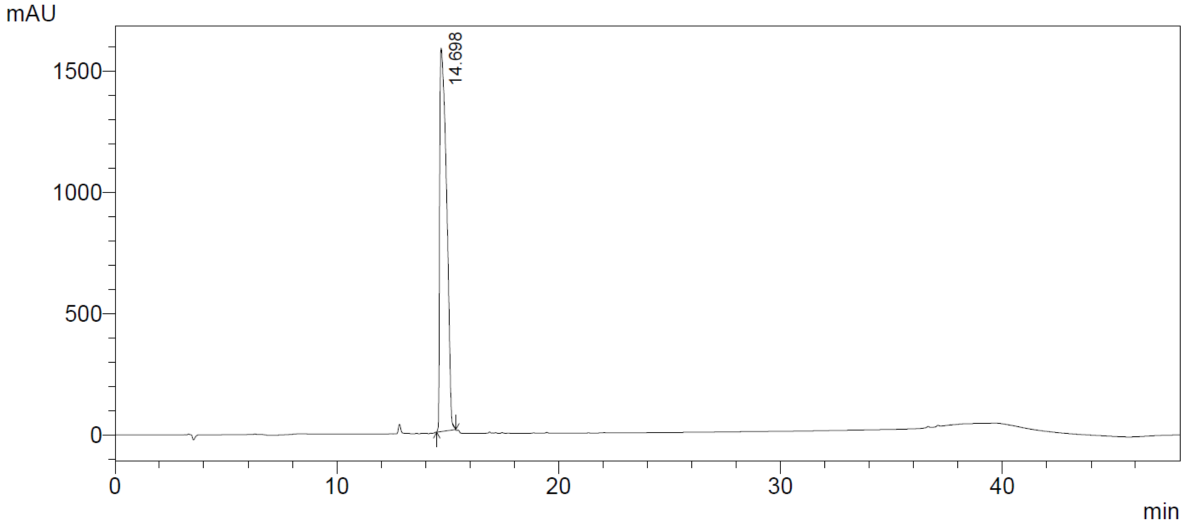

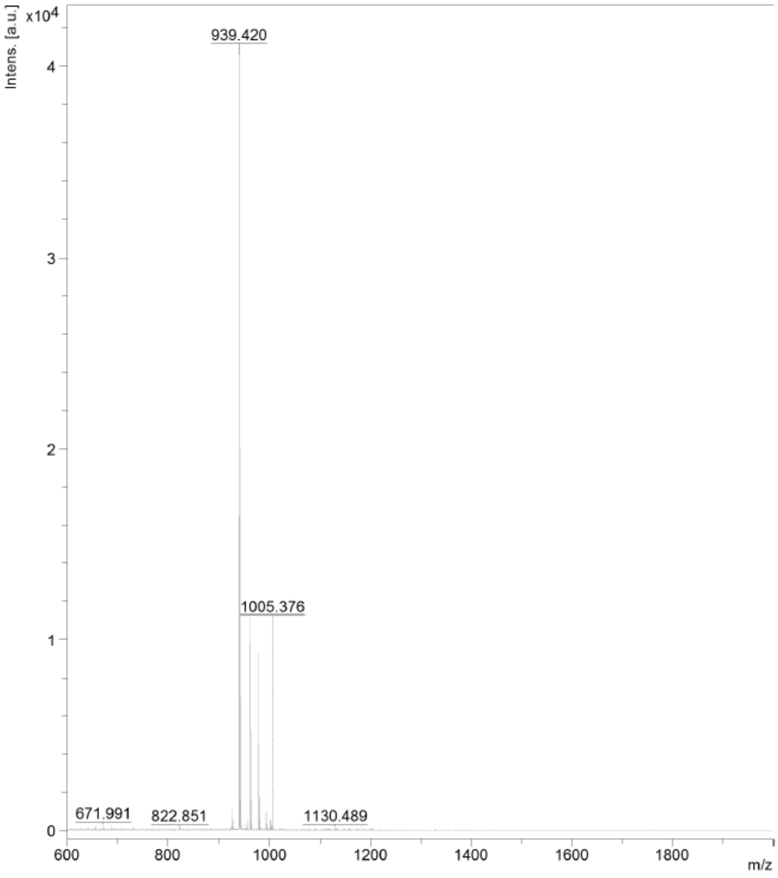

Asn-Gln-Trp-Ala-Ala-Gly-His-Leu-Met-NH2 , [Ala5]BN6-14

$[M+H]^+$ calculated 1026.185, found 1026.442

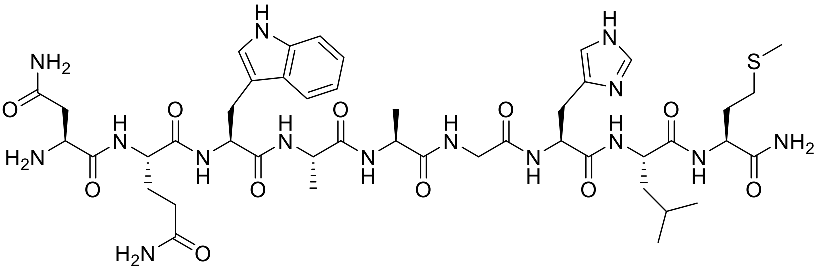

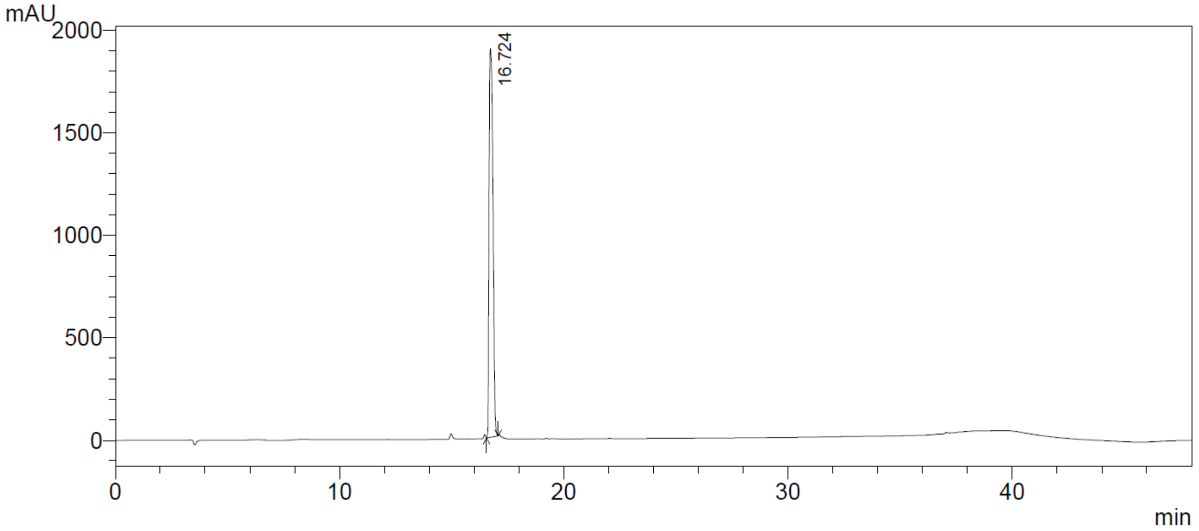

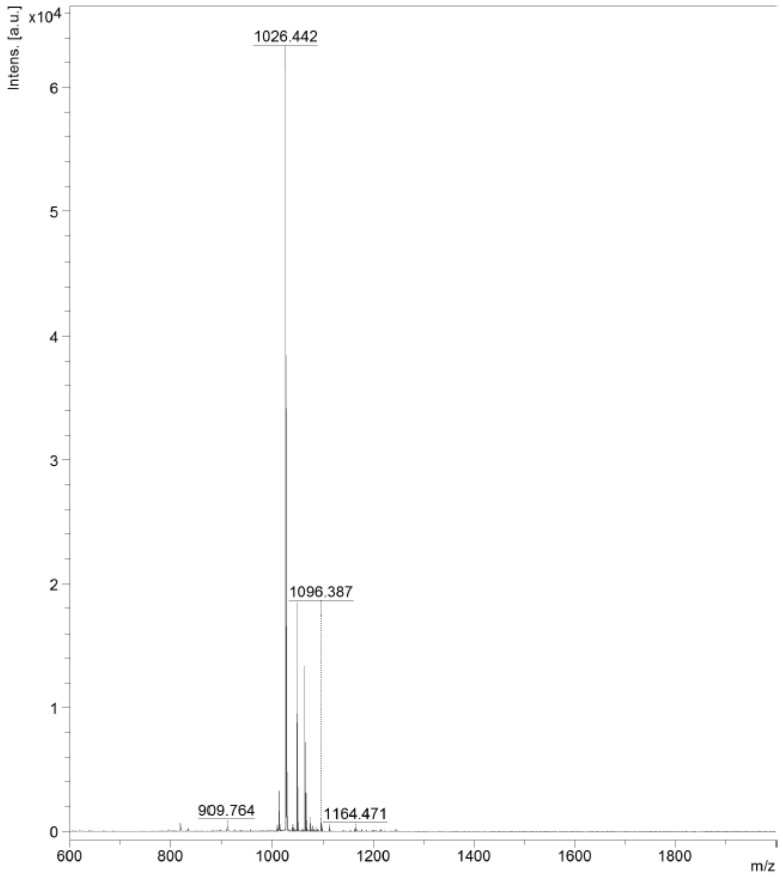

Asn-Gln-Trp-Ala-Val-Ala-His-Leu-Met-NH_2 , **[Ala^{6}]BN$^{6-14}$**

[M+H]$^{+}$ calculated 1068.266, found 1068.489

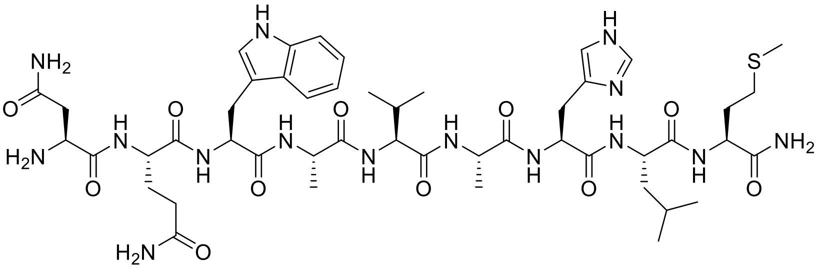

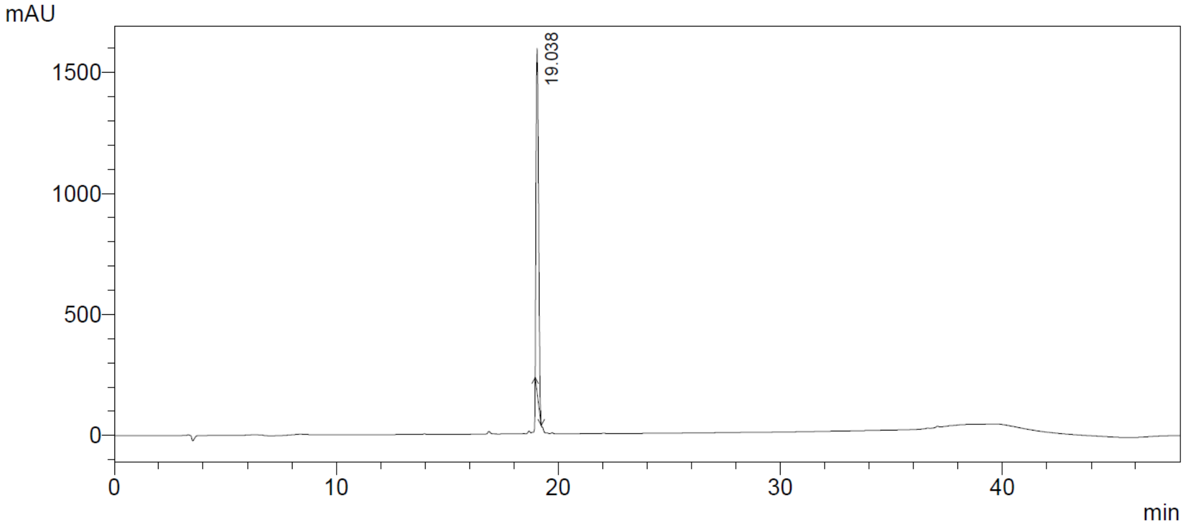

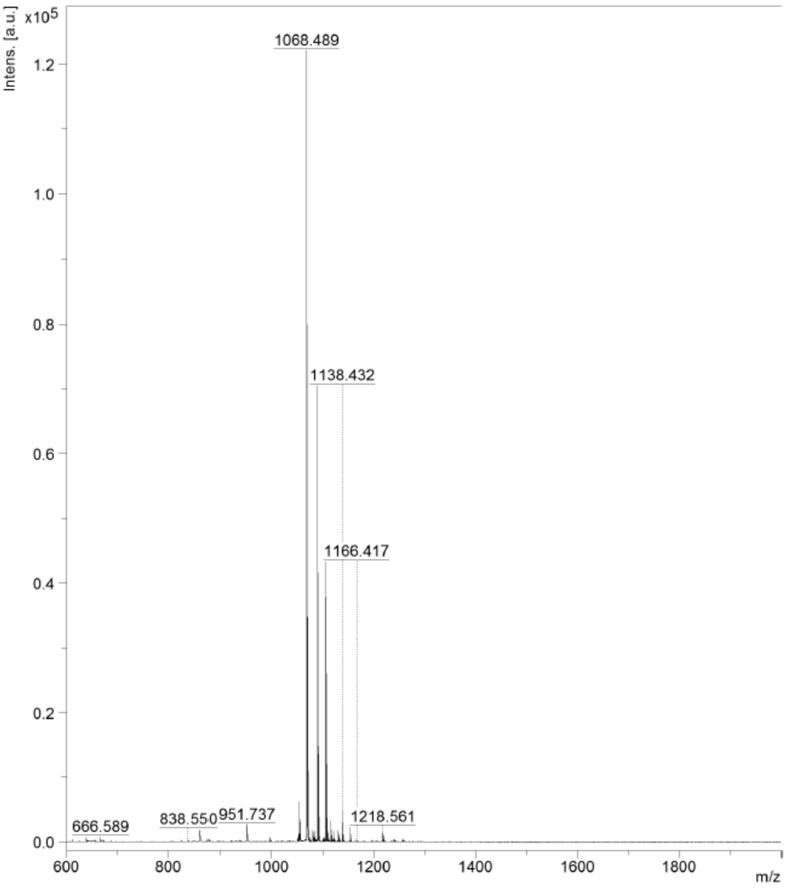

Asn-Gln-Trp-Ala-Val-Gly-Ala-Leu-Met-NH2, **[Ala7]BN6-14**

[M+Na]+ calculated 1010.485, found 1010.429

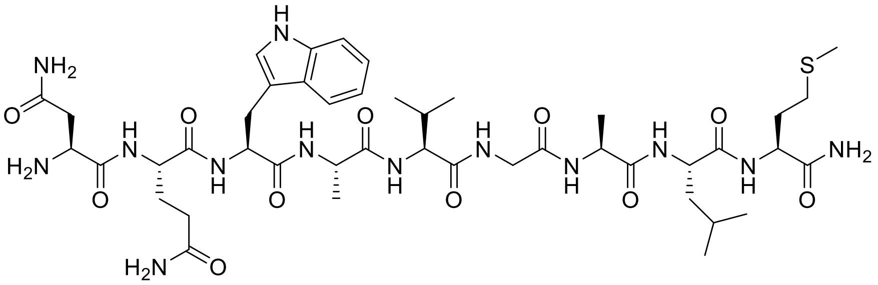

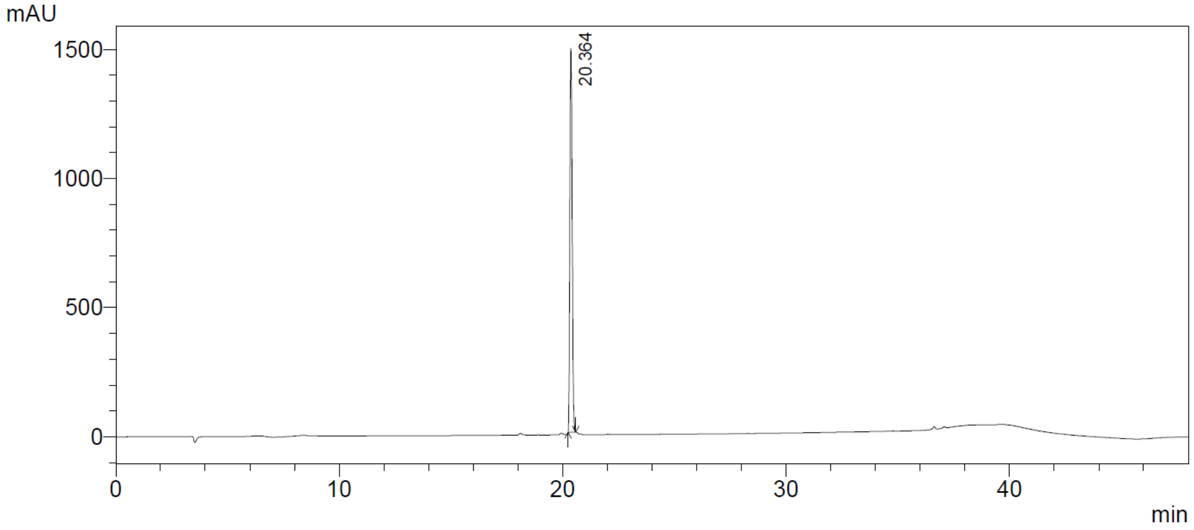

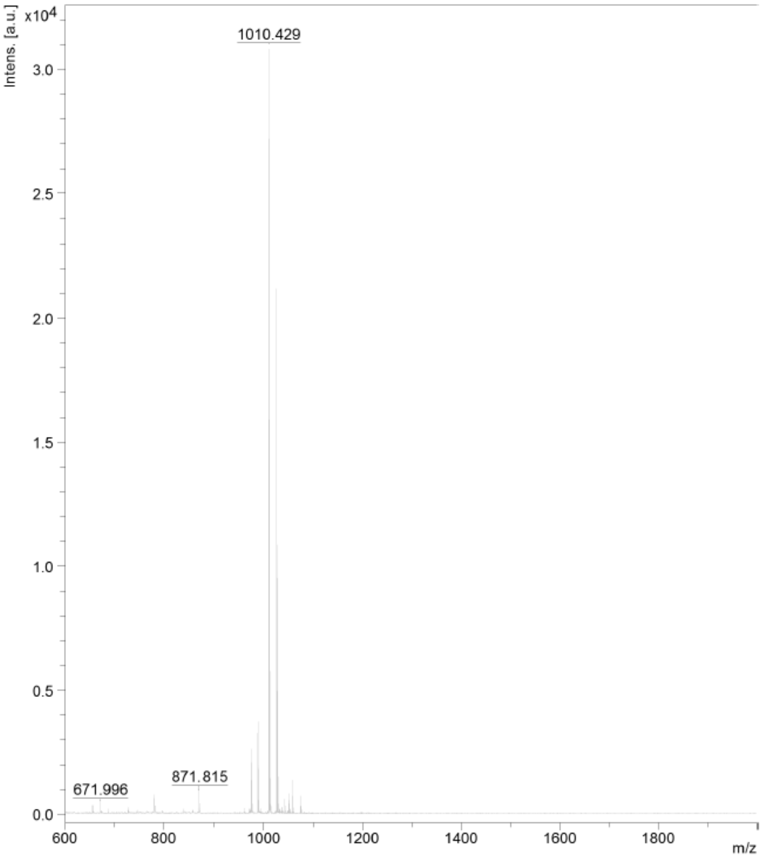

Asn-Gln-Trp-Ala-Val-Gly-His-Ala-Met-NH2, **[Ala[8]]BN[6-14]**

$[M+H]^+$ calculated 1012.158, found 1012.419

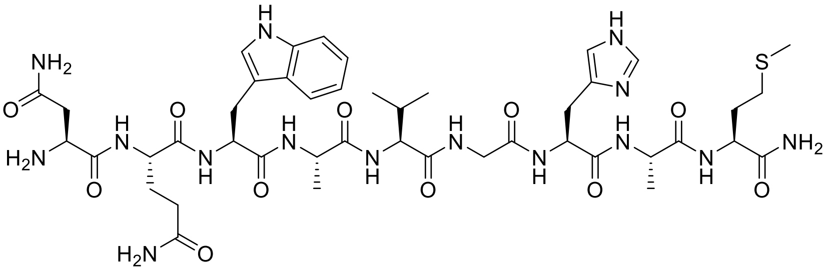

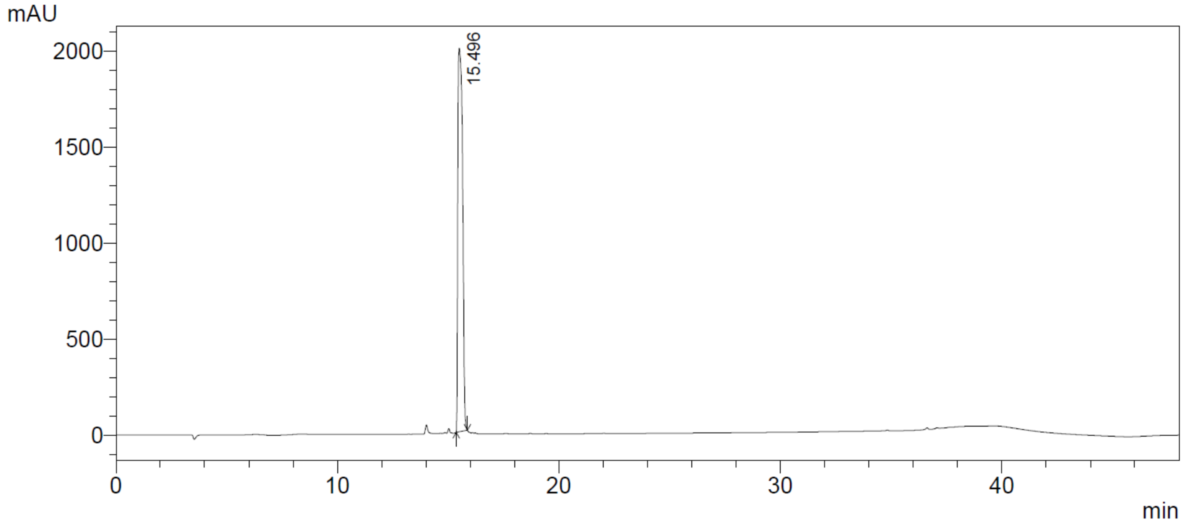

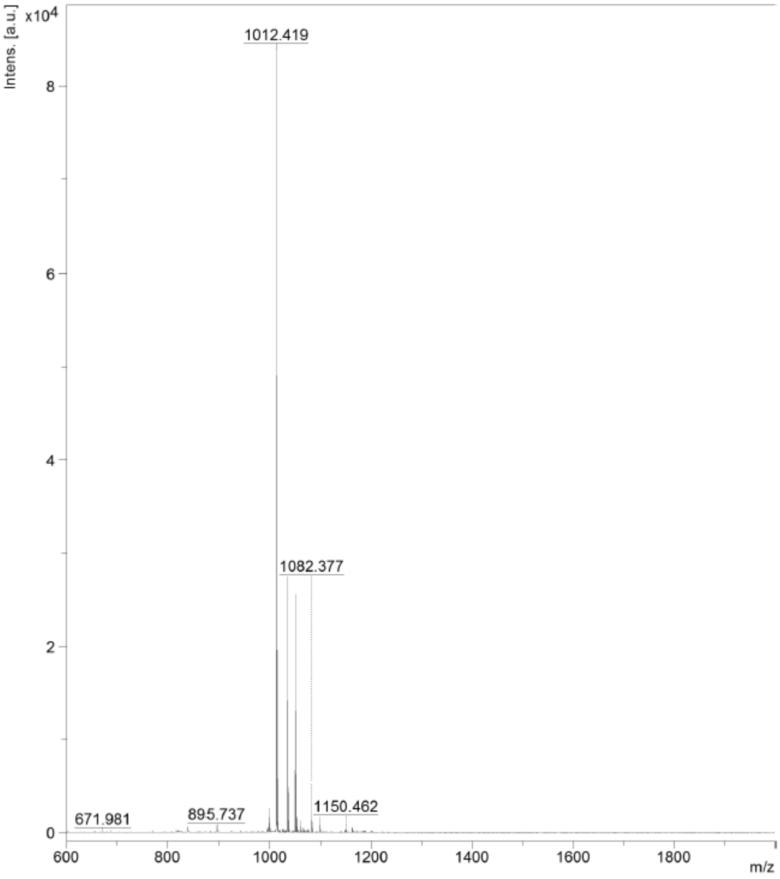

Asn-Gln-Trp-Ala-Val-Gly-His-Leu-Ala-NH2, **[Ala[9]]BN[6-14]**

$[M+H]^+$ calculated 994.125, found 994.468

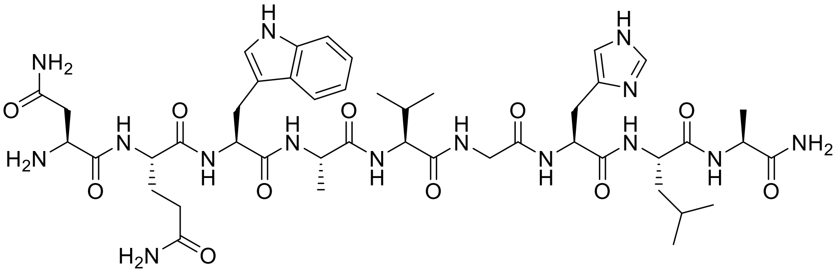

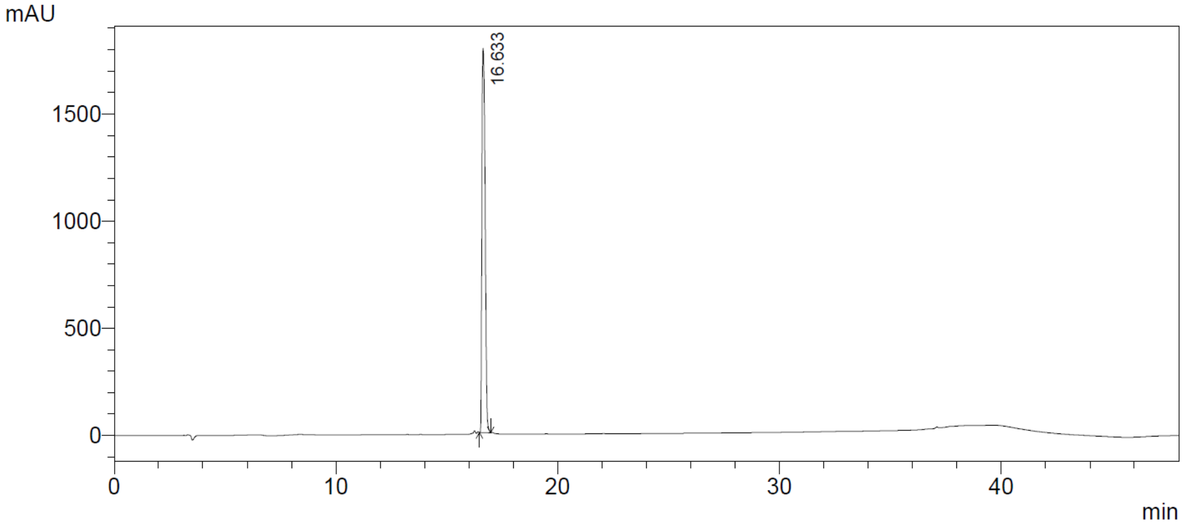

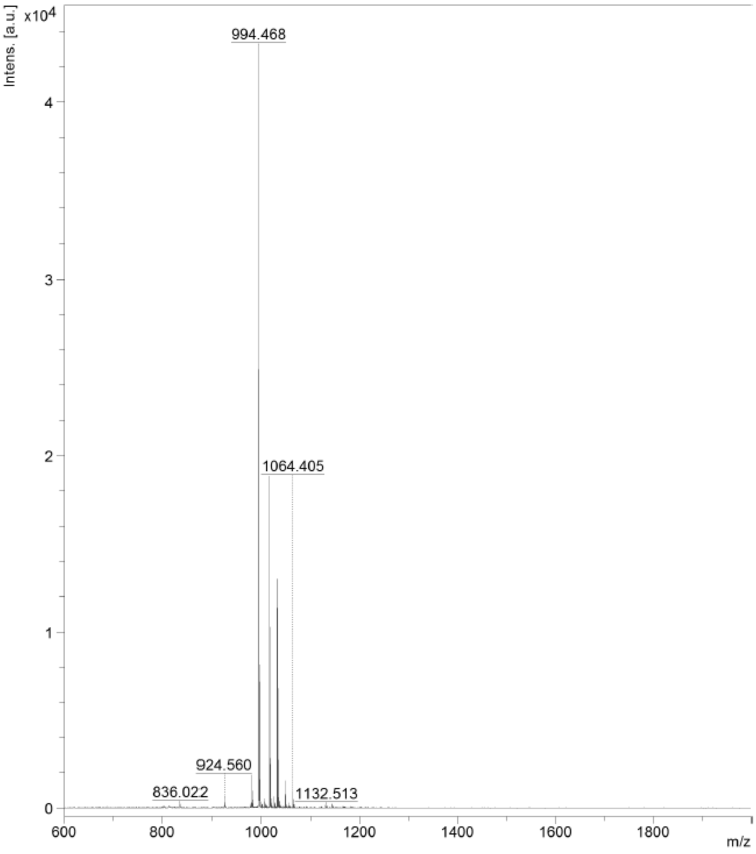

Supplement: Supplementary file 1 — jm1c00397_si_001.pdf [file jm1c00397_si_001.pdf]
